# Metabolic effects of mulberry branch bark powder on diabetic mice based on GC-MS metabolomics approach

**DOI:** 10.1186/s12986-019-0335-x

**Published:** 2019-01-31

**Authors:** Fan Qiu, Yu-Qing Zhang

**Affiliations:** School of Biology and Basic Medical Sciences, Soochow University, RM702-2303, No. 199, Renai Road, Dushuhu Higher Edu. Town, Suzhou, People’s Republic of China

**Keywords:** Diabetes mellitus, Gas-mass spectrometry, High fat diet, Metabolomics, Mulberry

## Abstract

**Background:**

Mulberry (*Morus multicaulis*) branch bark powder have showed effective hypoglycemic activity in our previous research. This study aims to explore the mechanism of protect effect on diabetes mice of mulberry branch bark as food supplement based on non-targeted GC-MS metabolomics’ platform.

**Methods:**

Animal model of double diabetes was established with high fat diet and Streptozotocin injection. Mice were fed with mulberry branch bark powder (MBBP) for five weeks to study its therapeutic effect. The metabolite feature of diabetes model and treatment group mice were characterized using a gas chromatography-mass spectrometry-based metabolomics, complemented with the biochemical evaluation, histological inspection, immunohistochemistry observations and enzyme protein detection.

**Results:**

A panel of endogenous metabolites were revealed that are relevant to disturbed metabolic processes among groups. The serum metabolic profiles were significantly different between the model group and treatment group. The manner of MBBP treatment showed to be significantly dose dependent and 20% MBBP treatment gain a relatively greater benefit than others. The metabolic disorders in model group include enhanced activation of the sorbitol pathway and galactose metabolite, increased activities of gluconeogenesis, fatty acid oxidation, proteins catabolism and attenuated activities of pentose phosphate pathway, glycolysis and aerobic oxidation pathways, internal synthesis of cholesterol, inositol production. MBBP treatment ameliorate these abnormal metabolize as revealed by differential metabolites comparing with that of model mice, such as decreasing the accumulation of ketone body, enhancing NADPH biosynthesis, partially reversing oxidative stress and energy metabolism disturbance.

**Conclusions:**

Mulberry branch bark had a re-balancing effect on the disturbed metabolic pathways in the diabetic mice. Based on the metabolic pathways network, oral administration of MBBP could ameliorate the hyperglycemia and hyperlipidemia symptoms in a global scale and restore the abnormal metabolic state to a near normal level in a dose dependent pattern.

**Electronic supplementary material:**

The online version of this article (10.1186/s12986-019-0335-x) contains supplementary material, which is available to authorized users.

## Background

Diabetes mellitus, characterized by elevated blood glucose levels, is a common chromic metabolic disease affecting millions individuals worldwide [1]. According to the International Diabetes Federation, the number of diabetic people was near 425 million in 2017 [2]. Unhealthy diet and sedentary lifestyle have further contributed to an increase in obesity and diabetes [3]. Two-thirds of people with diabetes live in urban areas and are of working age. The number of adults (20–79 years old) with diabetes mellitus in China has reached 114 million, which is still the largest number of diabetic patients in the world [2].

There are two types of diabetes, namely type 1 and type 2. Type 1 diabetes happens when the pancreas does not produce enough insulin to function properly [4]. Type 2 diabetes occurs when target tissues does not react to insulin effectively, namely insulin resistance [5]. However, there is an increase in the number of children and adolescents with a mixture of the two types of diabetes. Still, the lines between the two diseases can become blurred in more complicated cases, and double diabetes have been defined [6]. The new breed of diabetes, known as hybrid or double diabetes, in which an individual has the symptoms of both type 1 and type 2 diabetes were found associated with an increase in type 1 and type 2 diabetes in children and youths [7]. And evidence for the coexistence of insulin resistance and insulin deficiency in childhood-onset Type 1 diabetes adults has also been demonstrated by the insulin-glucose clamp technique [8]. The increasingly prevalence of complicated diabetes create a great need for alternative therapeutic strategies in people with double diabetes.

Mulberry (*Morus alba*) is a well-known medicinal plant and mulberry derived products in the form of mulberry fruit juice, mulberry leaves tea, root bark extracts and capsules are now commercially available as functional foods and dietary supplements [9]. Many studies have shown antioxidant [10], anti-inflammatory [11, 12], hypolipidemic [13], hypoglycemic [14], neuro-protective [15], antihypotensive [16] and antiviral activities [17] of mulberry. Different parts of mulberry revealed the presence of phenols, coumarins, flavonoids, alkaloids, saponins, polysaccharides and tannins, which are responsible for diverse pharmacological activities [18–20]. In our previous study, the branch bark of *Morus multicaulis* powder have showed effective hypoglycemic activity in mice which mainly contain prenylated flavonoids, Diels-Alder type adducts, stilbenes and polysaccharides [21–26]. And the evidence demonstrating beneficial effects of branch bark of mulberry against diabetes are still arising [27, 28].

In this study, a diabetes mice model with insulin secretion deficiency and insulin resistance induced by streptozotocin injection and high fat diet feeding was established to imitate hybrid diabetes in human. Three different dosages of mulberry branch bark powder were applied to feed diabetes model mice. A GC-MS based metabolomics approach was adopted to profile metabolites in serum samples collected from mice, complemented with the biochemical evaluation, histological inspection, verification of enzyme protein detection.

## Materials and methods

### Materials and preparation of MBBP

Streptozotocin (STZ, S0130) was purchased from Sigma-Aldrich Fine Chemicals, USA. All other chemicals and solvents were of analytical or HPLC grade. Methanol, acetonitrile, pyridine, n-hexane, methoxylamine hydrochloride(97%), BSTFA with 1% TMCS were purchased from CNW Technologies GmbH (Düsseldorf, Germany). L-2-chlorophenylalanine was from Shanghai Hengchuang Bio-technology Co., Ltd. (Shanghai, China).

The branches of the mulberry cultivar HuSang 32 from *Morus multicaulis* L. (*Morus alba var. multicaulis* (Perrott.) Loud.) were collected from the mulberry garden of Soochow University, Suzhou, China, in November 2016. The bark, which was peeled from the mulberry branches, was dried at 100 °C for 2 h, pulverized into powder twice, and passed through a 100-mesh sieve. The powder were weighted and mixed with standard diet to get 5, 10, 20% MBBP diets for mice.

### Animal procedures

Male C57BL/6 mice (18 ± 2 g) were obtained and housed in SPF Animal laboratory of the Laboratory Animal Research Center, Soochow University (Suzhou, China). The mice were maintained under controlled conditions (18–25 °C, 50–70% humidity, and a 12-h light/dark cycle, with free access to water and rodent chow). All procedures were approved by the Institutional Animal Care and Use Committee (IACUC) of Soochow University (number of animal license: 201704A034).

The mice were fed with a normal diet and a high fat diet containing 59% basic fodder, 20% sugar, 18% lard oil, and 3% egg yolk for five weeks. Then diabetes model was induced by intraperitoneal injection of freshly prepared citrate buffer (pH 4.5) solution of STZ (80 mg/kg) in high fat diet mice. Mice were randomly divided into five groups (*n* = 50): (1) normal diet supplied group (Normal), (2) high-fat diet and STZ injection group (Model), (3) 5% MBBP treated group (5%), (4) 10% MBBP treated group (10%) (5) 20% MBBP treated group (20%). The mice were then fed their individual diets for another five weeks.

At the end of the experiment, animals were lightly anesthetized with diethyl ether after 12 h of fasting, the blood and tissues were quickly removed from the mice. The tissues and organs of mice were stored at − 80 °C.Serum samples were obtained by stored at refrigerator at 4 °C overnight and then were centrifuged at 1026 g for 15 min at 4 °C.

### Intraperitoneal glucose tolerance test (IPGTT)

The mice were fasted for approximately 12 h, fasted blood glucose levels are determined before 20% glucose solution is administered by intra-peritoneal (IP) injection (2 g of glucose/kg body mass). Subsequently, the blood glucose level is measured with a portable OneTouch glucometer (from Johnson & Johnson Medical (Shanghai) Ltd., China) at 15, 30, 60, 90 and 150 min after glucose injection.

### Histopathological and immunohistochemical examination

The pancreas was quickly removed from the mice and fixed with 10% formalin solution. Tissue dehydration was performed with increasing concentrations of acetone. The sample was then cleaned with xylene and embedded in paraffin, and 3-μm thick slices were cut on an HM340E microtome, stained with hematoxylin and eosin (H&E) and insulin immunohistochemical staining, and the histocyte structure and expression of insulin protein were imaged under optical microscope (200×).

### Biochemical assay

The serum insulin levels were determined by Insulin Assay Kit (Nanjing Jiancheng Bioengineering Institute, Nanjing, China). The levels of glutathione peroxidase (GSH-Px), superoxide dismutase (SOD), malondialdehyde (MDA) in serum were measured using commercially available kits (Nanjing Jianchen Biotech Inc., China). The total cholesterol (CHOL), triglyceride (TG), high density lipoprotein cholesterol (HDLC) levels, low density lipoprotein cholesterol (LDLC), aspartate amino-transferase (AST), Uric acid (UA), Creatinine (Crea), Cholinesterase (CHE) levels in serum were assessed using a BS-800 Chemistry Analyzer (Mindray Medical International Ltd., ShenZhen, China).

### Sample preparation for GC/MS analysis

Serum samples stored at − 80 °C were thawed at room temperature. 50 μL of sample was added to a 1.5 mL eppendorf tube with 10 μL of 2-chloro-l-phenylalanine (0.3 mg/mL) dissolved in methanol as internal standard, and the tube was vortexed for 10 s. Subsequently, 150 μL of ice-cold mixture of methanol and acetonitrile (2:1, *v*/v) was added, and the mixtures were vortexed for 1 min, ultrasonicated at ambient temperature (25 °C to 28 °C) for 5 min, stored at − 20 °C for 10 min. The samples were centrifuged at 12000 rpm for 10 min at 4 °C. QC sample was prepared by mixing aliquots of the all samples to be a pooled sample. An aliquot of the 150 μL supernatant was transferred to a glass sampling vial for vacuum-dry at room temperature. And 80 μL of 15 mg/mL methoxylamine hydrochloride in pyridine was subsequently added. The resultant mixture was vortexed vigorously for 2 min and incubated at 37 °C for 90 min. 80 μL of BSTFA (with 1% TMCS) and 20 μL n-hexane was added into the mixture, which was vortexed vigorously for 2 min and then derivatized at 70 °C for 60 min. The samples were placed at ambient temperature for 30 min before GC-MS analysis.

### GC-MS analysis

Analyses were carried out on a GC-MS system (Agilent, model: 7890B) coupled with a mass selective detector (Agilent, model: 5977A). A DB-5MS fused-silica capillary column (30 m × 0.25 mm × 0.25 μm, Agilent J & W Scientific, Folsom, CA, USA) was utilized to separate the derivatives. The analysis was performed under the conditions described in following: Helium (> 99.999%) was used as the carrier gas at a constant flow rate of 1 mL/min through the column. The injector temperature was maintained at 260 °C. Injection volume was 2 μL by splitless mode, and the solvent delay time was set to 5 min. The initial oven temperature was 50 °C, ramped to 125 °C at a rate of 15 °C/min, to 210 °C at a rate of 5 °C/min, to 270 °C at a rate of 10 °C/min, to 305 °C at a rate of 20 °C/min, and finally held at 305 °C for 5 min. The temperature of MS quadrupole and electron impact ion source was set to 150 °C and 230 °C, respectively. The collision energy was 70 eV. Mass spectrometric data was acquired in a full-scan mode (m/z 50–450).

The QCs were injected at regular intervals (every 10 samples) throughout the analytical run to provide a set of data from which repeatability could be assessed.

Raw GC-MS data were converted to. CDF format by Agilent Chem Station software and imported into ChromaTOF (v 4.34, LECO, St Joseph, MI). The pretreatment process included the data baseline filtering and calibration of the baseline, peak alignment, deconvolution analysis, peak identification and integration of the peak area. Metabolites were qualitatived by the NIST and Fiehn database, which is lined to the ChromaTOF software. After internal standards and any known pseudo positive peaks, such as peaks caused by noise, column bleed and BSTFA derivatization procedure, were removed from the data set, the CSV file was obtained. The resulting data matrix which consists of sample code, peaks’ name, retention time, quant mass and peak intensities was further processed using Microsoft Execl 2010. Total chromatographic area normalization was applied to reduce the deviation between each sample. Finally, the normalized dataset was imported into SIMCA 14 (Umetrics, Sweden) for multivariate statistically analysis.

### Western blot

Protein was isolated from hepatic tissue using lysis buffer enrich with phosphatase and protease inhibitors (Keygen biotech, Nanjing, China) and quantitated using Bradford protein quantification kit (Yeasen, shanghai, China). Equal amounts of protein were loaded and separated on sodium dodecyl sulphate polyacrylamide gels and then incubated with specific antibodies. The following primary antibodies were used: GLK (bs-1796R), PEPCK1 (bs-4972R), PFK1 (bs-3982R), CPT1A (bs-2047R) from Bioss Inc. (Beijing, China); G6P (sc-398,155) from Sant Cruz biotecnology (CA, USA); HMGCR (BM4908) from Boster biological technology (Wuhan, China). Images were captured with G: BOX chemiXR5 imaging System (Syngene, Cambridge, UK) and analyzed with Gel-Pro32 software.

### Data processing

The data were analyzed using Origin 9.1 software. The data of model groups was compared with that of normal group and the data of MBBP treat group was compared with that of model groups. Statistical significance was performed using unpaired 2-tailed Student’s *t*-test or ANOVA, and results with a *P*-value < 0.05 were considered to be statistically significant.

Multivariable analysis including principal component analysis (PCA) and orthogonal projection to latent structures-discriminant analysis (OPLS-DA) analysis was performed by Simca 14. Pathway analysis was performed with Metaboanalyst 4.0 (http://www.metaboanalyst.ca).

## Results

### Assessment of diabetes animal model and therapeutic efficacy of MBBP

**Fig. 1:**
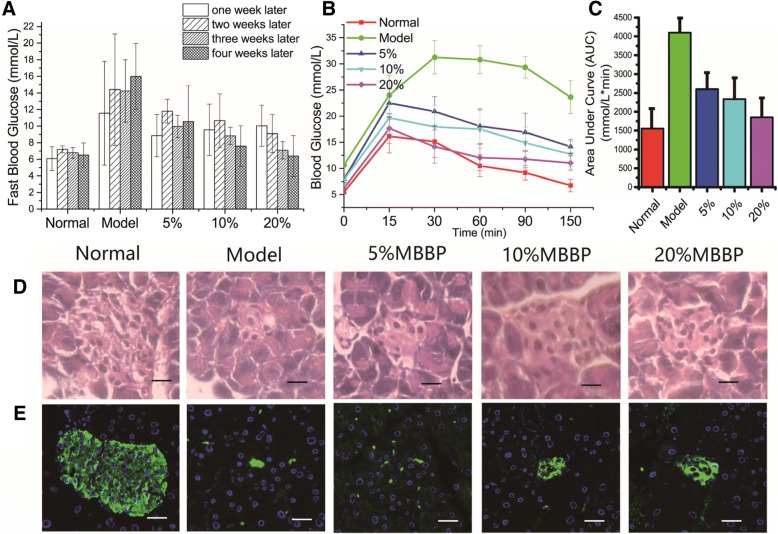
**a**, The fast glucose levels after STZ injection of model group and MBBP treat group; **b**, Intraperitoneal glucose tolerance test (IPGTT). The mice were fasted for 12 h, and the baseline blood glucose was measured at 0 min, and then 2 g glucose/kg body weight was injected intraperitoneally. Glucose levels were tested at regular intervals of 15, 30, 60, 90 and 150 min; **c**, Quantification of the area under the curve (AUC) from the IPGTT; **d**, Histopathological examination of pancreas by H&E staining; **e**, Immunofluorescence examination of insulin in pancreas by Alexa Fluor 488 (green)

**Fig. 2:**
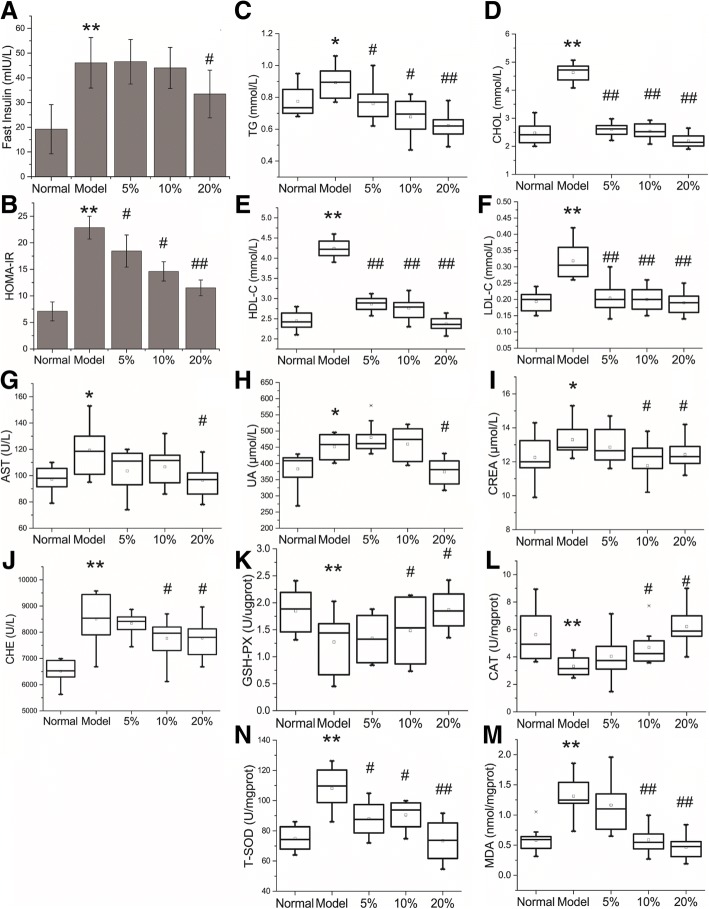
**a**, the fast insulin level of mice after the diabetes mice were treated five weeks; **b**, the index of Homeostatic Model Assessment of Insulin Resistance (HOMA-IR), HOMA-IR = Glucose (mmol/L)╳Insulin (mU/L)/22.5; C-N, boxplots for values of TG (**c**), CHOL (**d**), HDL-C (**e**), LDL-C (**f**), AST (**g**), UA (H), CREA (**i**), CHE (**j**), GSH-PX (**k**), CAT (**l**), T-SOD (**m**), MDA (**n**). *p < 0.05 and ** *p* < 0.01 model group vs. normal group, # p < 0.05 and ## p < 0.01 MBBP treatment group vs. model group

After STZ injection and five weeks of high fat diet feeding, significant diabetes symptoms were observed in model group mice. Food intake and fluid intake in model group was significantly higher than other group. Mean body weight gain increased with high fat diet and decreased after STZ injection sharply (Additional file 1: Figure S1). The fasting blood glucose levels of mice continue to be high (> 7.8 mmol/L) in model group after STZ injection (Fig. 1a). Intraperitoneal Glucose Tolerance Test (IPGTT) measures the clearance of intraperitoneally injected glucose load from the body. As shown in Fig. 1b, the blood glucose levels of model group mice were significantly increased following the injection of glucose and cleared slower in comparison with normal group mice. The total area under the curve (AUC) of blood glucose levels in model group between 0 to 150 min were significantly higher than normal group (Fig. 1c). In addition, the data of MBBP treat group indicate that MBBP significantly decreased fasting blood glucose levels and improved glucose tolerance in a dose dependent manner when compared with model group (Fig. 1a-c). The fast insulin were also detected (Fig. 2a) and the homeostasis method assessment of insulin-resistant was calculated using the following formula: HOMA-IR = Glucose (mmol/L) × Insulin (mU/L)/22.5 (Fig. 2b). It is a terrific way to reveal the dynamic between your baseline (fasting) blood sugar and the responsive hormone insulin [29]. The HOMA-IR value of model group was significantly higher than that of normal group, which indicates significant insulin resistance. The presence and extent of insulin resistance decreased with MBBP treatment as shown by decreased HOMA-IR value in MBBP treat groups.

### Histopathological and Immunohistochemical assessment

The pancreas can secrete insulin and is an important organ for regulating blood glucose. Islets of Langerhans are functional units of the endocrine pancreas [30]. Figure 1d shows the pathological changes in the pancreas of five group mice by H&E staining. Compared with normal group, the islets of Langerhans become smaller and the cells lost their normal structure, showing swelling, granular degeneration, local necrosis, and some cells have become pyknotic, karyorrhexic and karyolytic. With the treatment of different doses of MBBP for five weeks, pancreatic cell necrosis and pancreatic damage were reduced and pancreatic islets retained larger volume. Figure 1e shows the insulin secretion changes in the pancreas of five group mice by Alexa Fluor 488 (green). In normal group, the islets shows a normal structure with insulin-secreting *β*-cells. The model group mice exhibited markedly damaged insulin secretion in comparison with the normal group. With the MBBP treatment, the pancreas regained the capacity of insulin secretion, but not as better as the pancreatic cell structure recovery.

### Serum biochemical analysis

To investigate the effects of MBBP on lipid metabolism, the levels of TG, CHOL, HDL-C, and LDL-C were determined (Fig. 2c-f). High fat diet caused a marked elevation of CHOL accompanied by a significant HDL-C and LDL-C increase and a slight rise of TG level in the mice of model group. With the treatment of MBBP, the metabolic imbalance were relieved.

Aspartate amino-transferase (AST), which were biochemical markers for assessing hepatic injury, was significantly elevated (*P* < 0.05) in the mice of model group. Uric acid (UA) and Creatinine (Crea), as biochemical index of kidney dysfunction [31], were also increased in model group and decreased in MBBP treat group. The serum concentration of Cholinesterase (CHE) was also observed rise up in model group and declined in MBBP treat group (Fig. 2g-j).

To explore the antioxidant capacity of mice fed with the MBBP diet, the levels of MDA, GSH-PX, T-SOD and CAT were determined (Fig. 2k-n). The levels of MDA, the oxidative stress marker, exhibited a significant increase in the model group (P < 0.05) compared with normal group. MBBP feed treatment significantly decreased the enhanced levels of MDA in model mice. The model groups had a notable reduction of GSH-PX and CAT levels, which could also be significantly reversed by MBBP. However, the level of T-SOD were increased in model group compared with normal group. With the treatment of MBBP feed, the level of T-SOD decreased in mice.

### Metabolite identification

TIC spectra of serum samples are shown in Additional file 1: Figure S2. A total of 321 metabolites in serum extracts were identified by querying NIST and Fiehn database. Detailed information about the metabolites is listed in Additional file 2: Tables S1.

### Multivariate analysis of GC-mass spectral data of all groups

**Fig. 3:**
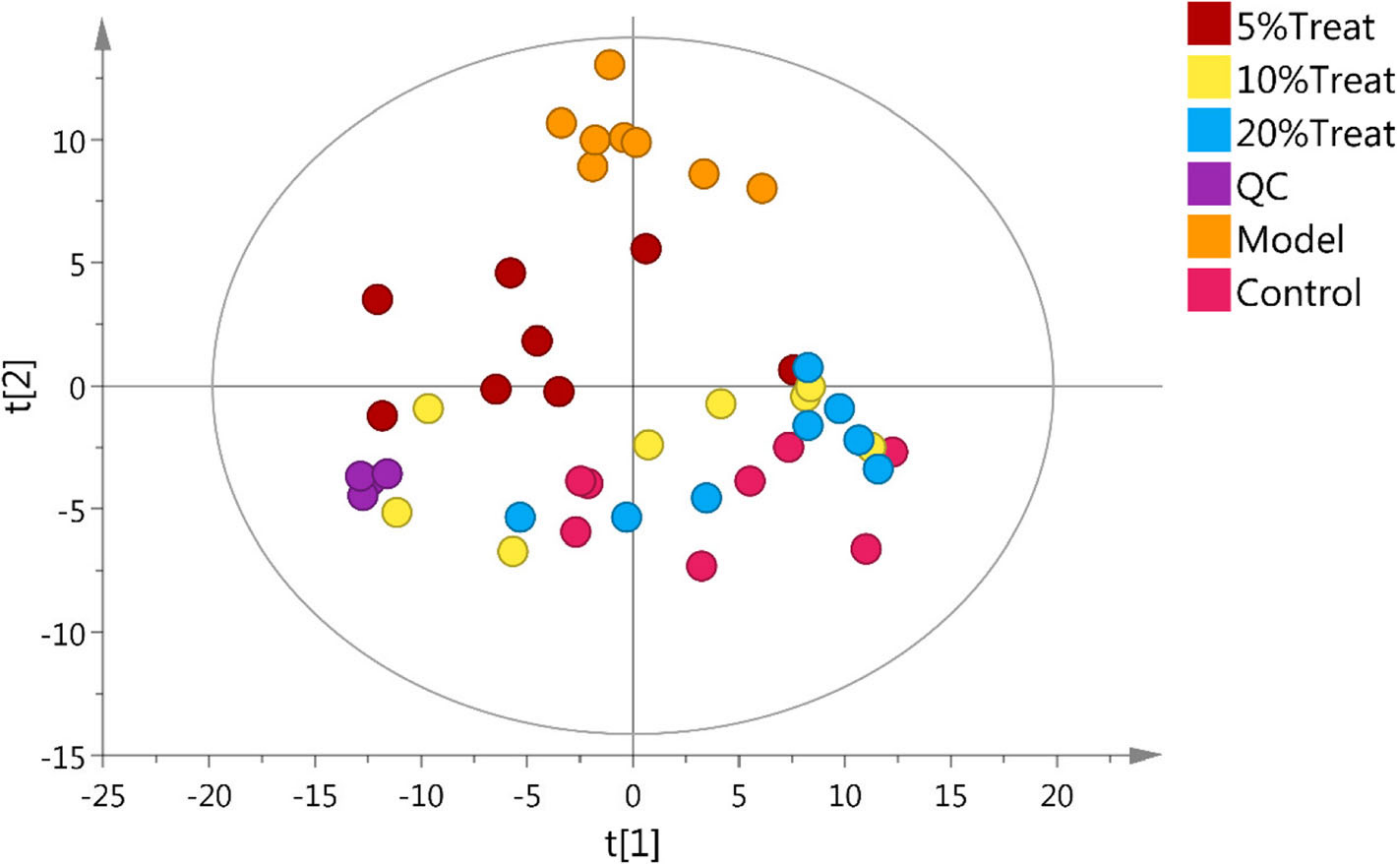
the PCA score plot of all groups, 7 components, R2X [1] = 0.186, R2X [2] = 0.0945, Ellipse: Hotelling’s T2 (95%), R2X (cum) = 0.546, Q2(cum) = 0.226

There is a lot of GCMS data and it is hard find any trends or outliers by analyzing the raw data. Therefore PCA analysis were done after unit variance scaling (Fig. 3). The PCA model has seven components with R^2^X (cum) = 0.546, which points to a stable model. Q2 is low, 0.226, indicating that although patterns are found they are not conclusive. Each point represent one sample in the score plot. The quality control (QC) samples in the PCA score plot overlapped indicate samples behaved stable for the duration of the run. There are no outliers in the score and DModX plot shows no deviating observations. Normal group and Model group were completely separated and far away. The MBBP treat groups close to normal group with a dose dependent pattern. This demonstrate severe metabolic disturbance induced by high fat diet and STZ could be ameliorated after MBBP feed in mice.

**Fig. 4:**
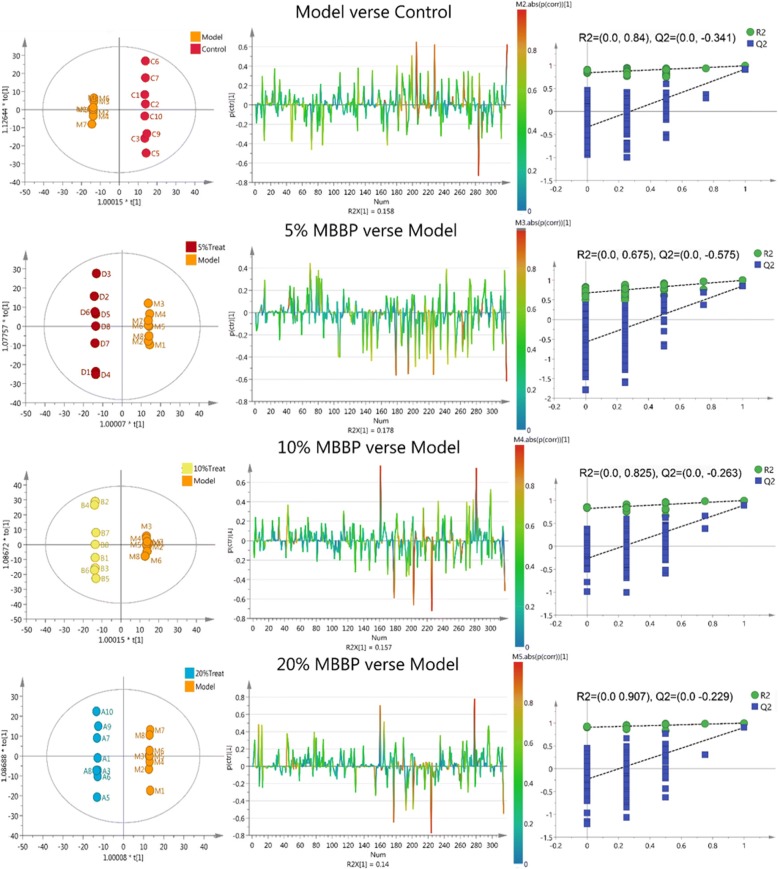
OPLS-DA score plots and S-line plots for discriminating the metabolite and corresponding permutation test (200 times) obtained from GC-MS

Orthogonal partial least squares discriminant analysis (OPLS-DA) was subsequently employed to explore intergroup difference in Fig. 4. In the OPLS-DA score plots of the serum extracts, Normal group was completely separated from the model group (R2Y (cum) = 0.999, Q2 (cum) =0.808), demonstrating metabolic disturbance exist in model group in response to high fat diet and STZ injection. Moreover, a well-separated tendency from model group and MBBP treat groups were similarly displayed, suggesting that a different metabolic pattern existed between the model group and MBBP treat groups. The parameters R2Y (cum) and Q2 (cum) value of 0.999 and 0.8 were considered to be a good fitness and predictability of the constructed OPLS-DA model respectively. To validate the model and avoid over fitting, permutation test (*n* = 200) were presented in Fig. 4, the analytical platform presented excellent performance in stability and repeatability, and could be exploited in subsequently metabolomics research.

### Differential metabolite analysis

Differential metabolites contributing to the separation were identified using variable importance in the projection (VIP) value, fold change values of metabolites and the corresponding *p* values. In general, a threshold of VIP > 1 was consider as the relevant metabolites for interpreting the discrimination, fold change value ≥1.5 or ≤ 0.667 were supposed to be obvious up-regulated or down-regulated in concentration, and Student’s t test p value set to 0.05 (*p* < 0.05) was believed to be a significant difference. Based on the strategy, a total of 46 discriminating metabolites resulting from model group were selected when compared with normal group (Table 1). There are 43, 43 and 40 discriminating metabolites resulting from 5, 10, 20% MBBP treat group respectively when compared with model group (Additional file 2: Table S2–S4).

**Table 1:** Differential metabolites in response to model group vs. normal group

No	Metabolites	VIP*	t-test P*	FC*	Trend
1	Sophorose	1.948	4.41E-06	2.139	Up
2	Thiogalactopyranoside	1.953	5.43E-06	2.072	Up
3	Glucose	1.711	7.87E-05	2.069	Up
4	Maltose	1.976	1.66E-06	2.054	Up
5	Sorbitol	1.917	1.69E-05	1.988	Up
6	D-(glycerol 1-phosphate)	1.501	0.001135	1.905	Up
7	3,6-Anhydro-D-galactose	1.765	9.95E-06	1.786	Up
8	Fructose	1.078	0.03948	1.758	Up
9	Tagatose	1.763	0.00056	1.728	Up
10	D-Fructose 1,6-bisphosphate	1.879	9.97E-06	0.602	Down
11	Maltotriose	1.315	0.028018	0.507	Down
12	1,5-anhydroglucitol	1.395	0.006196	0.480	Down
13	Sucrose	2.023	0.005275	0.163	Down
14	Erythrose	2.136	7.17E-10	0.103	Down
15	Gluconic lactone	1.403	0.002208	0.071	Down
16	Galactose	1.920	2.88E-05	0.047	Down
17	L-Malic acid	1.590	0.001329	0.656	Down
18	3-hydroxy-3-methylglutaric acid	1.498	0.010145	3.371	Up
19	Palmitoleic acid	1.794	0.000802	2.483	Up
20	Linolenic acid	1.822	7.01E-05	1.501	Up
21	Linoleic acid	1.986	9.87E-08	0.559	Down
22	Linoleic acid methyl ester	1.223	0.006099	0.315	Down
23	Monoolein	1.310	0.000797	0.292	Down
24	Zymosterol	1.023	4.05E-05	0.230	Down
25	Hydrocinnamic acid	1.137	0.006299	0.207	Down
26	Arachidonic acid	1.958	4.18E-05	0.198	Down
27	3-Hydroxyanthranilic acid	1.499	0.023418	2.390	Up
28	Cycloleucine	1.823	0.000221	2.025	Up
29	Glutamine	1.583	0.000371	1.630	Up
30	Methionine	1.610	0.003739	0.634	Down
31	Canavanine	1.117	0.040176	0.625	Down
32	O-phosphonothreonine	1.480	0.002276	0.624	Down
33	4-aminobutyric acid	1.099	0.048405	0.576	Down
34	Citrulline	1.924	7.34E-06	0.495	Down
35	Sarcosine	1.268	0.003764	0.411	Down
36	4-hydroxyphenylethanol	2.078	3.57E-11	3.620	Up
37	Hippuric acid	1.027	0.049162	0.151	Down
38	Taurine	2.074	5.83E-08	0.391	Down
39	O-phosphorylethanolamine	1.829	0.000744	3.523	Up
40	Maleimide	1.361	0.00019	3.055	Up
41	5-Aminoimidazole-4-carboxamide	1.115	0.028222	6.135	Up
42	Inositol	2.169	1.325E-10	0.000	Down
43	Allantoic acid	1.675	0.000228	0.418	Down
44	Kyotorphin	1.245	0.000396	0.294	Down
45	5,6-dihydrouracil	1.213	0.013668	0.045	Down
46	Trans,trans-Muconic acid	1.364	0.029647	0.000	Down

* Variable importance in the projection (VIP) was obtained from OPLS-DA with a threshold of 1.0; P value was calculated from student’s test. Fold change (FC) was calculated from the mean value between Model and Normal group

The significant metabolites include sugars (glucose, fructose, fructose 1,6-bisphosphate, galactose, gluconic lactone etc.), sugar alcohols (sorbitol, 4-hydroxyphenylethanol, 1,5-anhydroglucitol), organic acids (malic acid, 3-hydroxy-3-methylglutaric acid, allantoic acid, 4-aminobutyric aicd, 3-hydroxyanthranilic acid), fatty acid (palmitoleic acid, linolenic acid, arachidonic aicd), amino acids (glutamine, citrulline, sarcosine etc.) and others (taurine, O-phosphorylethanolamine, kyotorphin, 5,6-dihydrouracil etc.). Xenobiotics originated from plant and syntheitic drugs such as trehalose, guaiacol, quinic acid, phloroglucinol, beta-Sitosterol, Atrazine-2-hydroxy, 1-Aminocyclopropanecarboxylic acid, albendazole were excluded from all metabolite list.

### SUS shared and unique analysis

**Fig. 5:**
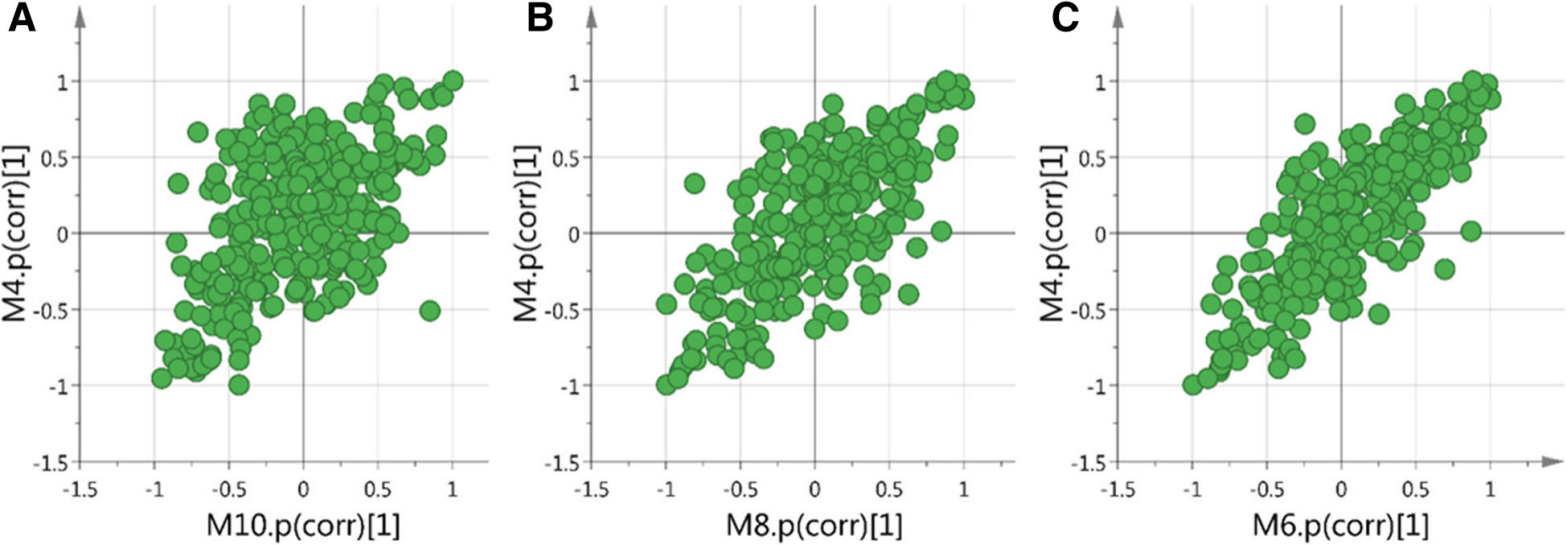
Shared and unique structure plot (SUS plot) of serum correlating the OPLS-DA models of Model verse 5% MBBP treatment (M10) and Models versus Normal (M4) (**a**); Model verse 10% MBBP treatment (M8) and Models versus Normal (M4) (**b**); Model verse 20% MBBP treatment (M6) and Models versus Normal (M4) (**c**)

The SUS plot is used when there is a three group investigation. Two OPLS-DA models construct a SUS plot (Fig. 5). Each point represent one variable (metabolite) in the score plot. The OPLS-DA have the model names M4 (Model vs. Normal), M6 (Model vs. 20% MBBP treatment), M8 (Model vs. 10% MBBP treatment) and M10 (Model vs. 5% MBBP treatment). Take plot C in Fig. 5 as an example, on the horizontal line we see unique structure. Variables far out to the left and to the right change in the M6 model but do not change in the M4. On the vertical line we also see unique structure. Variable in the top and in the bottom change in the M4 model but do not change much in the M6. On the diagonal we see shared structure. In the upper right corner, the up regulated variables for both normal and MBBP treatment mice can be found. In the lower left corner the variables that are down regulated for both normal and MBBP treatment mice can be found. The left upper corner shows the structures that are up regulated in normal group and down regulated for the MBBP treatment mice and the lower right corner shows the variables that are up regulated in the MBBP treatment mice group and down regulated in the normal mice group. There is obvious tendency for MBBP treatment mice similar to normal mice in a dose dependent way for the scatter points tend to be in a diagonal line.

**Fig. 6:**
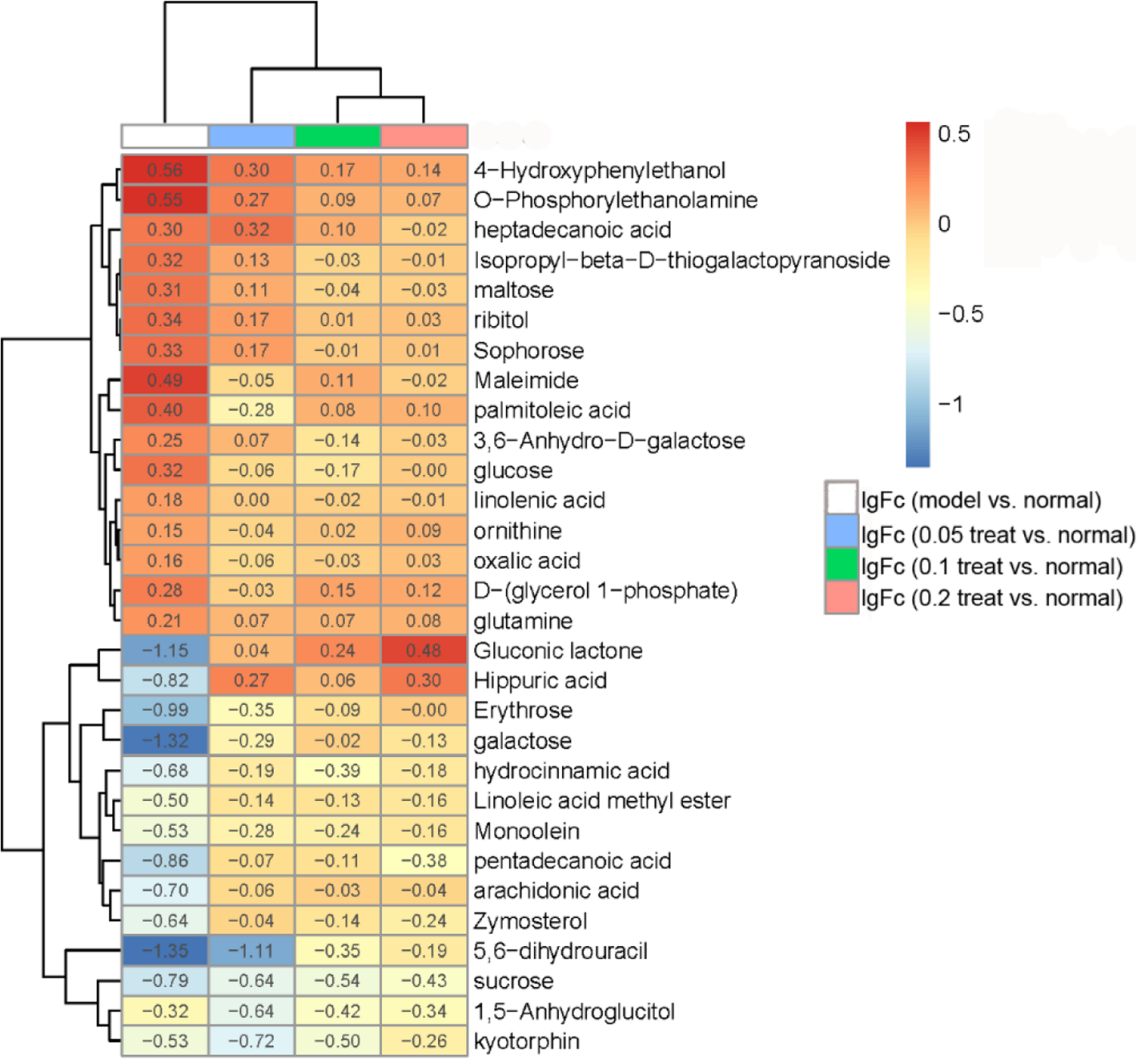
Heat map of changes in the levels of 30 metabolites in response to the MBBP treatment concentration compared to model group. The log10 fold change in ratios of the means for each group are shown in plot. Red indicates an up-regulation effect, whilst blue indicates a down-regulation effect

The details of this trend were improvably visualized in heat map by 30 metabolites in Fig. 6. The ratio of the peak areas between model group and normal group, MBBP treatment group and normal group were calculated. The logarithms of the ratio of means for each group (model vs. normal, 5% MBBP vs. normal, 10% MBBP vs. normal, 20% MBBP vs. normal) were used to plot a heat map in R. Log ratio = log (means of a metabolite area in model or MBBP treatment group / means of a metabolite area in normal group). Log ratio values above 0 indicate an up-regulation of metabolites and below 0 indicate a down regulation of metabolites compared to normal ones.

### Metabolite pathway analysis

**Fig. 7:**
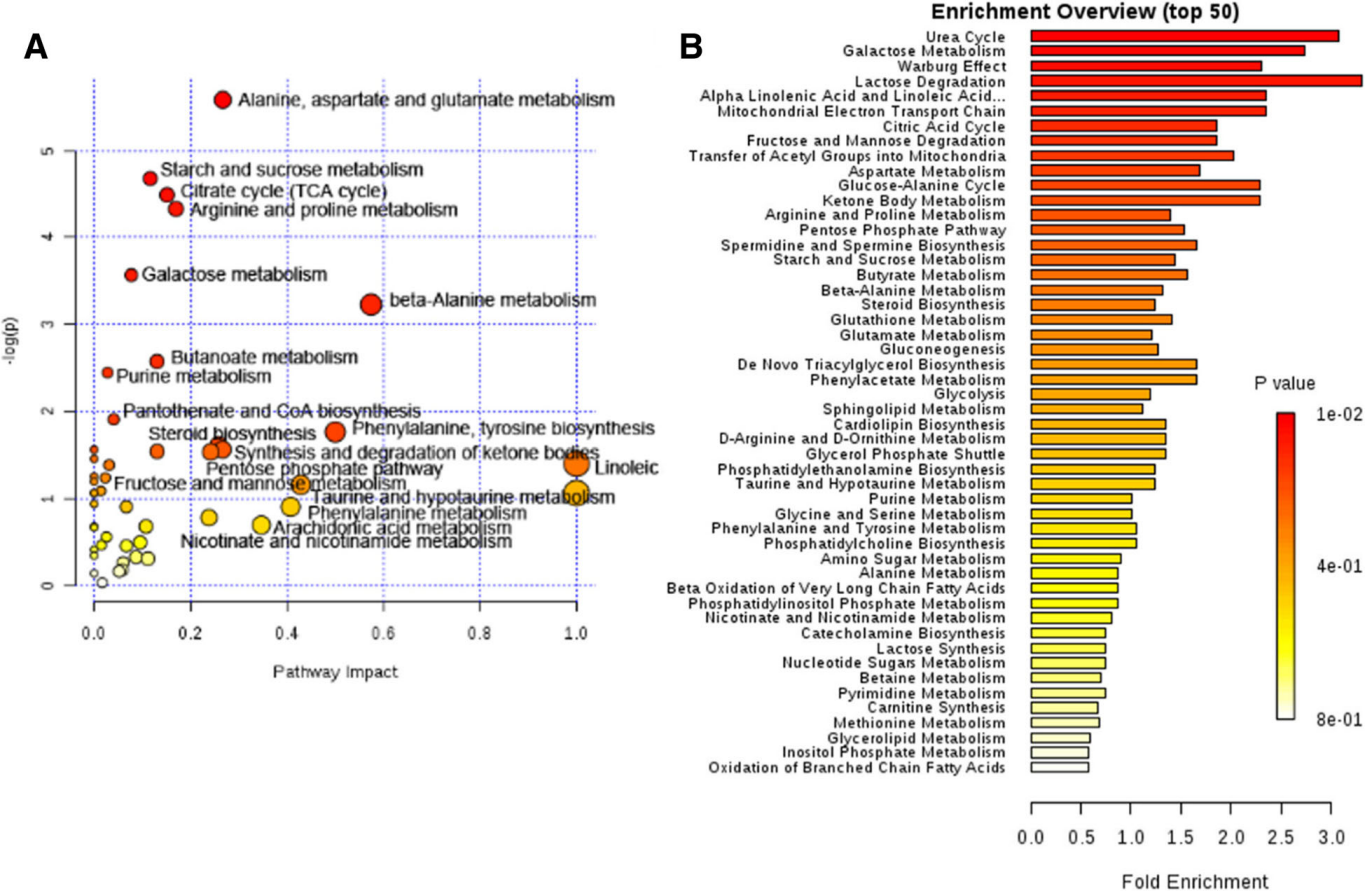
Disturbed metabolic pathway in Model group mice as visualized by bubble plots (**a**) and enrichment overview (**b**)

**Fig. 8:**
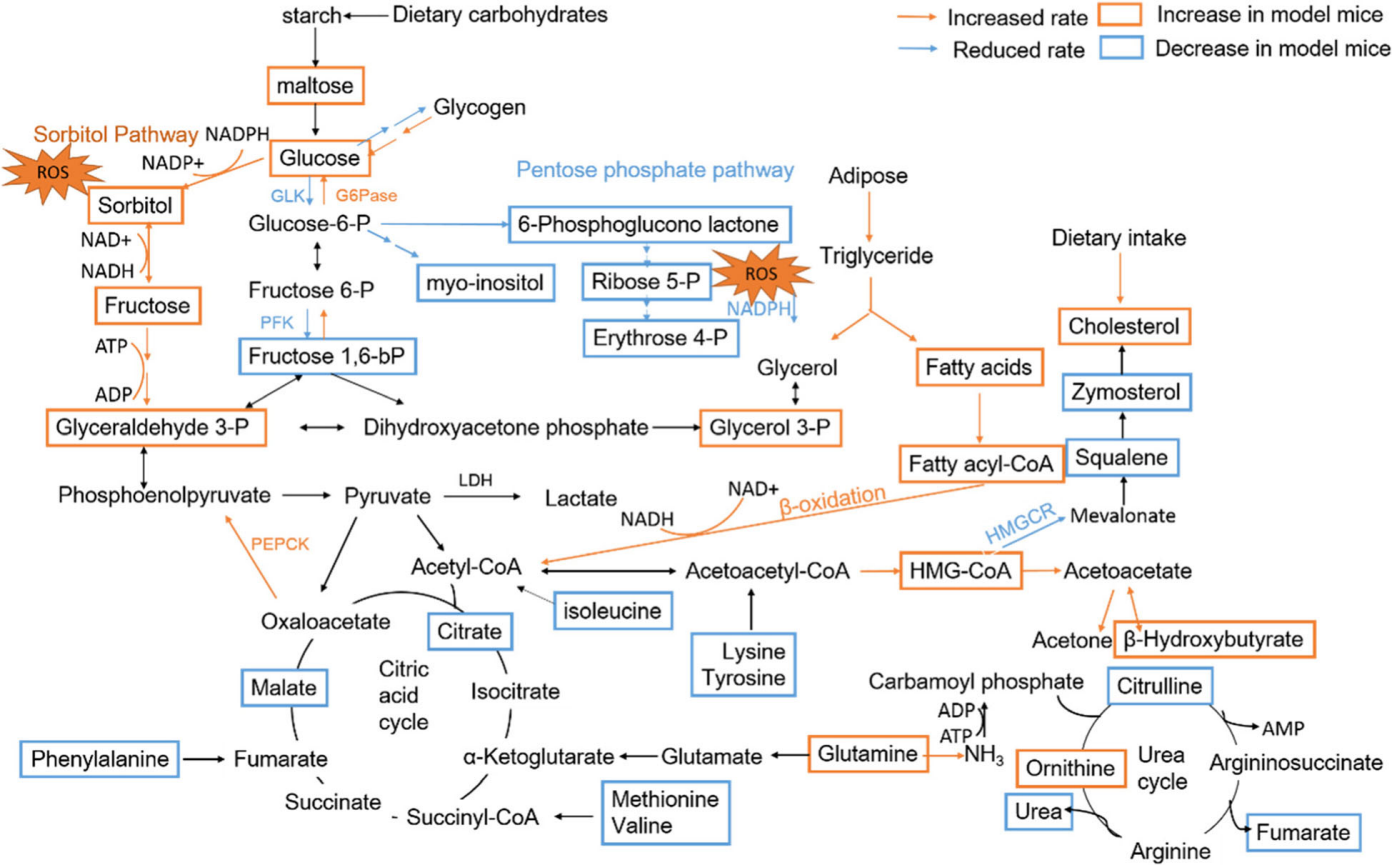
Schematic diagram of the disturbed metabolic pathways detected by GC-MS analysis, showing the interrelationship of the differential metabolites

Significant metabolites selected above were subjected to enrichment analysis and pathway topology analysis using MetPA (http://www.metaboanalyst.ca) to explore biologically meaningful metabolic patterns and the most impacted pathways. Compared to normal mice, the significant perturbed metabolic pathways in serum of high fat diet and STZ injection were determined (Fig. 7). These metabolic pathways were further analyzed in the KEGG database (http://www.kegg.jp/) and SMPDB (http://smpdb.ca/), five meaningful metabolic pathways were summarized, including carbohydrate metabolism, lipid metabolism, protein metabolism, energy metabolism, oxidative stress. Based on the above pathway analysis, a map of the diabetes related metabolic pathways was constructed (Fig. 8).

## Discussion

In this study, complemented with serum biochemistry, histopathology and immunohistochemistry, a GC-MS based metabolomics was adopted to investigate the specific metabolic events in diabetes model mice and the amelioration effect of MBBP. Compared with normal mice, mice in model group showed high glucose level, insulin resistance, significantly changed level of liver and kidney function index, revealing typical diabetes symptoms due to severely impaired *β*-cells of pancreas. MBBP could markedly improve the pancreas function of diabetes mice for at least one month MBBP diet, consistent with the results of biochemical involved in lipid metabolism and oxidative stress. MBBP treatment mice showed to be more resistant to STZ injection injury, which is in consistent with several previous reports. One of the reason might be ingestion of mulberry fiber and active ingredients removes the pathogenic factor of high fat and high carbohydrate diet. OPLS-DA and SUS plot analysis of GC-MS data from serum revealed metabolic perturbations in carbohydrate metabolism, lipid metabolism, amino acid metabolism, energy metabolism, oxidative stress, and s series of potential biomarkers for high fat diet and STZ induced diabetes.

### Carbohydrate metabolism

Compared with normal mice, levels of glucose, maltose, sucrose, sorbitol, tagatose were significantly increased; the level of 1, 5-anhydroglucitol, erythrose, galactose, gluconic lactone, fructose 1, 6-bisphosphate, maltotriose in model group were significantly decreased. These changes were reversed in MBBP treatment group in dose dependent way. These metabolites were related to galactose metabolism, lactose degradation, fructose and mannose degradation, pentose phosphate pathway, starch and sucrose metabolism, gluconeogenesis, glycolysis metabolism in pathway overview plot (Fig. 7).

**Fig. 9:**
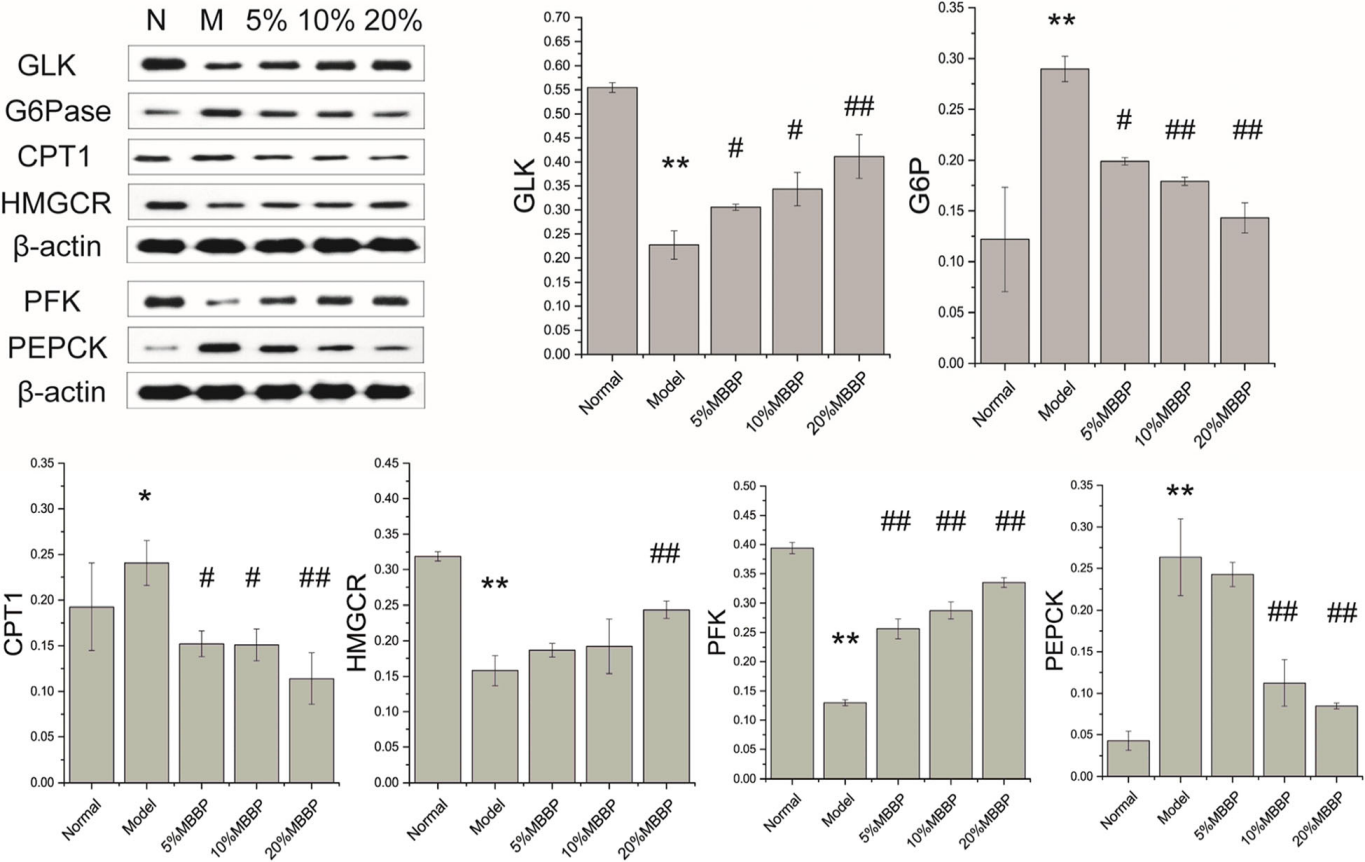
Protein expression as determined by western blotting

Glucose, fructose, and galactose are the main hexoses absorbed from the gastrointestinal tract, derived principally from dietary starch, sucrose, and lactose, respectively. Fructose and galactose are converted to glucose, mainly in the liver [32]. In model group, the high level of maltose and glucose indicate decreased glucose utilization in model mice. Reduced activity of glucokinase (GLK) and Phosphofructokinase-1 (PFK-1) in western blot assay underscore the reduced rate of glycolysis [33]. Under the effect of glucagon, increased activity of enzymes of pathway of gluconeogenesis such as Glucose-6-phosphatase (G6Pase), Phosphoenolpyruvate carboxykinase (PEPCK) increase the rate of gluconeogenesis (Fig. 9). Similarly, glucagon plays a major role in the absence of insulin function [32], glycogen metabolism enzyme activities are altered by glucagon triggered phosphorylation cascade. Glycogenesis was inhibited due to reduced activity of glycogen synthase, glycogenolysis was stimulated due to increased activity of phosphorylase [34]. This aggravated the increase of serum glucose level in model group mice. In MBBP treatment mice, this situation has been improved according to these metabolite concentration and enzyme activity in reverse direction.

Decreased concentration of 6-Phosphoglucono lactone, Ribose 5-phosphate, Erythrose 4-phosphate indicate inhibited pentose phosphate pathway in model group mice. The pentose phosphate pathway is an alternative route for the metabolism of glucose. It was suppressed due to reduced activity of glucose-6-P dehydrogenase enzyme as that is under the influence of insulin. This pathway does not generate ATP but has two major functions: (1) the formation of NADPH for synthesis of fatty acids and steroids and counter the damaging effects of oxygen radicals. (2) the synthesis of ribose for nucleotide and nucleic acid formation [35]. Inhibited pentose phosphate pathway provide little NADPH, this was responding to the effects of oxidative stress [36], fatty acid oxidation in model group. According to the increased metabolite concentrations (6-Phosphoglucono lactone, Ribose 5-phosphate, Erythrose 4-phosphate) in pentose phosphate pathway in MBBP treatment groups, it was found that MBBP treatment could boost the recovery of this pathway and maintain a reducing atmosphere.

Hyperglycemia increases glucose metabolism via the sorbitol pathway. Intracellular glucose is predominantly metabolized by phosphorylation and subsequent glycolysis, but when increased, some glucose is converted to sorbitol [37]. Sorbitol in liver is converted to fructose in the presence of sorbitol dehydrogenase. In this polyol pathway, decreased NADPH/NADP^+^ and increased NADH/NAD^+^ result in redox imbalance and oxidative stress through reduced level of glutathione and increased reactive oxygen species concentrations [38]. The increased fructose metabolize lead to excess consumption sequesters inorganic phosphate and depletes ATP levels. These adverse situation were alleviated with declined level of sorbitol and fructose in MBBP treatment group mice.

In the molecular pathway of inositol, after insulin links with the cell, inositol-second messengers are produced [39]. In model group mice, the low inositol concentration were in agreement with loss of insulin function. However, in MBBP treatment group mice, the level of inositol substantially increased and greater than normal. Second messenger based on myo-inositol regulate glucose intake increasing the activity of glucose transport proteins [40]. Inositol acts as a precursor for PI3K kinase. Inositol both produces and activates PI3K kinase, which were necessary for normal cell glucose metabolism [39]. This bring into correspondence with increased rate of glycolysis in MBBP treatment group mice when compared with that of mode group mice.

### Lipid metabolism

Increased concentration of palmitoleic acid, lauric acid and glycerol phosphate in model group indicate rapid mobilization of triglycerides from adipose tissue leading to increased levels of plasma free fatty acids. The free fatty acids are taken up by numerous tissues and metabolized to provide energy [41]. The activity of carnitine palmitoyl transferase I increased in model group in western blot suggested increased fatty acid oxidation, for the enzyme required for the transport of fatty acyl-CoA’s into the mitochondria where they are subject to oxidation for energy production [42]. Correspondingly, the declined level of monoolein, a product in the synthesis of triglyceride, indicate there is absence for fatty acids flowing to synthetic pathway. Mitochondrial oxidation of fatty acids generates acetyl-CoA which can be further oxidized in the TCA cycle. But in hepatocytes the majority of the acetyl-CoA is not oxidized by the TCA cycle, it is metabolized into the ketone bodies, acetone, acetoacetate and β-hydroxybutyrate. The high level of 3-hydroxy-3-methylglutaric acid and significantly ketone and butyrate metabolism mirrored the increased ketone bodies. This classic biochemical changes in diabetes were still alleviated represented by the reverse fatty acid level and CPT1 activity in MBBP treatment group.

The amount of cholesterol carried by LDL-cholesterol (LDL-C) were observed to be high in model group mice by biochemistry assay. The high level of cholesterol in serum was mainly from the high fat diet feed in model mice. The declined level of squalene and zymosterol, as cholesterol synthesis intermediates [43], showed decreased cholesterol biosynthesis in model group mice. The decreased activity of HMG-CoA reductase, which is the rate-controlling enzyme of mevalonate pathway to produces cholesterol corroborated this fact. In addition, the high level of cholesterol promotes the increase of HDL-cholesterol and the decrease of linoleic acid in serum for the circulation of linoleic acid and HDL-cholesterol [44]. Due to the declined level of linoleic acid, the level of downstream metabolites, arachidonic acid and prostaglandin E2 were also observed to be redueced. It is noteworthy linolenic acid, the first step product in the metabolism of linoleic acid [43], was observed to be high level. It suggested another way to generate linolenic acid was enhanced, which was the cleavage of phospholipids into their constituent fatty acids by phospholipase A2. The enhancement of phospholipid catabolism is also confirmed by the increase of other products such as O-phosphorylethanolamine (a polar head group of sphingolipid), palmitoleic acid (long chain fatty acid). In this series of lipids pathways, these metabolites mentioned above in MBBP treatment group changed in the opposite direction as well.

### Protein metabolism

Insulin deficiency or tolerance has lead complex biochemical changes in glucose metabolism and lipid metabolism, it still be the key for protein metabolism. Insulin has a global effect on protein metabolism, increasing the rate of protein synthesis and decreasing the rate of protein degradation [45]. Thus, the catabolism of protein was enhanced in model group mice for insulin resistance. The level of lysine, tyrosine and isoleucine declined slightly (VIP > 1, FC < 1, model vs. normal) as ketogenic proteins which give rise to acetyl-CoA and ketone bodies. The level of methionine, valine and phenylalanine also decreased slightly (VIP > 1, FC < 1, model vs. normal) as glucogenic proteins which give rise to succinyl-CoA and fumarate and production of glucose. Some protein transformation products such as taurine from cysteine and γ-aminobutyric acid from glutamine also decreased in model group.

During the process of catabolism of protein, ammonia was transferred in muscle and other various tissues, the level of glutamine and alanine increased in model group for they carry ammonia to liver in circulatory system [46]. In liver, alanine can be trasaminated again producing pyruvate that can be used for gluconeogenesis and glutamine will release ammonia to form urea by urea cycle. The level of ornithine was also observed increased in model group as evidence for the release of ammonia from other tissues. However, the declined level of citrulline and urea indicate a breakdown in the urea cycle which consume ammonium ion, CO_2_ and ATP. This may be due to ATP deficit via depletion of TCA cycle in mitochondrion [47] or activation of nitric oxide synthase whereby nitric oxide production is competitively favored over urea production [48].

In MBBP treatment group, we found the process of protein degradation has been reversed and urea cycle defects disappeared based on the backward changing data in MBBP group.

### Energy metabolism

The most notable change in the metabolic profile of diabetes model is reduced glucose oxidative metabolism and increased fatty acids β-oxidation metabolism. The main fuel of the body inclined to be fatty acid instead of glucose as fuel to produce energy in model mice. Oxidation of fatty acid lead to excess production of NADH and H^+^ and high NADH/NAD+ ratio in mitochondria [42]. This slows three NAD dependent oxidation (isocitrate dehydrogenase, α-ketoglutarate dehydrogenase, malate dehydrogenase) in TCA cycle [43]. And reduced TCA cycle flux in model group were observed according to the level of malic acid (FC = 0.656), succinic acid (FC = 0.792), fumaric acid (FC = 0.761), citric acid (FC = 0.812) when compared with normal group. The inhibition by high levels of NADH without ATP may lead to unmatched rate of citric acid cycle to meet energy demand [49]. In addition, the reason for suppressed TCA cycle in model group may be due to non-availability of oxaloacetate as it is channeled towards glucose production.

The increase of creatinine in serum were observed in model group which was degraded from phosphocreatine. This suggested more high-energy phosphate bond in phosphocreatine has been transferred to form ATP for energy demand and confirmed energy deficit state in mode group mice.

In MBBP treatment group, the TCA cycle come to rescue, the balance of energy supply inclined to be normal judging by the level of malic acid, succinic acid, fumaric acid, citric acid and creatinine in MBBP treatment groups.

### Oxidative stress

With the reduction of NADPH supplied in pentose phosphate pathway, organism would fail to meet their anabolic demands and combat oxidative stress. Besides, the sorbitol production in polyol pathway intensified the insufficiency of NADPH and up-regulation of the NADH oxidase complex in model group. NADPH is crucial for the regeneration of GSH via the glutathione disulphide (GSSG) reductase, following O_2_^.-^ is generated from the NADPH oxidase reaction or mitochondrial electron transport chain (ETC) are dismutated by the mitochondrial Mn-SOD or cytosolic Cu-Zn-denpendent SOD [50]. NADPH is also utilized to regenerate GSH after GPx-catalysed reduction of H_2_O_2_, if the latter is not reduced by catalase (CAT) [50, 51]. By maintaining a reducing atmosphere (a high ratio of NADPH to NADP and a high ratio of reduced to oxidized glutathione), they can prevent or undo oxidative damage to cell membrane, lipids, proteins and other sensitive molecules [5, 52].

Several biochemical parameters which were measured to reflect the status of oxidative stress confirmed the oxidative imbalance in model group mice. Comparing with the normal group, the level of malonaldehyde (MDA), as a lipid peroxidation product, increased in model group; the activity of antioxidant enzyme Glutathione peroxidase (GSH-Px) and catalase (CAT) also declined in model group; the activity of total-SOD were found a little higher in model group. These demonstrated reactive oxygen species (ROS) generation and oxidative stress occurring in the model mice. MBBP treatment could ameliorate these abnormal parameters as revealed by decreased concentration of MDA and increased activity of GSH-Px and CAT, when compared with model mice.

## Conclusions

Diabetes is a chronic disease with serious metabolic disturbances in carbohydrate, protein [50] and fat metabolism [30] arising due to insulin deficiency or insulin dysfunction. In this study, a panel of endogenous metabolites were revealed that are relevant to disturbed metabolic processes among groups, the metabolite feature of diabetes model and treatment group mice were characterized and the interrelationship of the identified metabolites were analyzed. The metabolic disorders in model group include enhanced activation of the sorbitol pathway and galactose metabolite, increased activities of gluconeogenesis, fatty acid oxidation, proteins catabolism and attenuated activities of pentose phosphate pathway, glycolysis and aerobic oxidation pathways, internal synthesis of cholesterol, inositol production. MBBP treatment ameliorate these abnormal metabolize as revealed by differential metabolites comparing with that of model mice.

In addition, there are sufficient data to correlate the magnitude of the abnormalities in glucose metabolism with those of fat (or protein) metabolism. In model group, the ketone bodies increase to compensate for impaired glucose metabolism as energy providers for tissues; increased fatty acid oxidation compensates glucose oxidation defects to produce ATP. However, there is no alternative pathway for pentose phosphate pathway to produce NADPH, an important reducing power in cytoplasm, and this results in an imbalance in redox state. These abnormal metabolism is substantially reversed in MBBP treatment group. This suggested the mulberry branch bark play a therapeutic role through multiple targets or key targets. The hypoglycemic effect of MBBP were related to their regulation of oxidative, increase of inositol, amelioration of disordered metabolism which were influenced by insulin or not. It open up avenue of investigation of signaling pathways of these process for more detailed studies of the inherent molecular mechanism and targets.

The observed beneficial effects of mulberry branch bark are postulated to be brought about by the activity of various identified specific constituents (deoxynojirimycin, polysaccharides, morusin, mulberroside A, quercetin, phytosterols, chlorogenic acid etc.) and some nonspecific compounds. Its nutrient composition (fibers, vitamins, minerals, amino acids) may also play a role and be indispensably as therapeutic nutrition. Because this is in line with the principle of balanced diet in the treatment of diabetes mellitus. Together with the results in this study, mulberry branch bark have shown the potential of treating diabetes mellitus as a supplementary food or raw material in functional food. However, when oral administration of mulberry branch bark powder are applied in people with diabetes, the dosage of other chemotherapeutic medicine may be decreased. The exact dosage of mulberry branch bark when combined with other medicine or not in diabetic people needs to be further studied.

As a research based on untargeted metabolomics which provide insights into fundamental biological processes of diabetic mice fed with mulberry branch bark in this study, the relevant information have been extracted from the large-scale data in a holistic manner. However, untargeted metabolomics casts a much more indiscriminate net, considering all metabolite information in an experiment to examine multiple sets of profiles without bias [53]. For each disturbed metabolic pathway, a statistical ranking were given, but this is not a biological importance ranking. Various metabolite changes can be related in the metabolic network and can be verified each other. The most challenging further study is to trace these metabolic changes and clarify the upstream molecules such as mRNA and DNA etc. In addition, it is worth noting that GC-MS also can not cover all the metabolites of organisms and there are dynamic limitations for one-time detection in metabolomics platform.

## Additional file

Additional file 1: **Figure S1.** The effect of high fat diet, STZ injection and MBBP treatment on the body weight of mice. **Figure S2.** Total ion current (TIC) chromatogram of serum of mice of Normal group (a), Model group (b), 5% MBBP treat group (c), 10% MBBP treat group (d), 20% MBBP treat group (e) obtained from GC-MS analysis. (DOCX 589 kb)

Additional file 2: **Table S1.** Peaks identified in TIC chromatogram of serum from five groups after peak alignment. **Table S2.** Differential metabolites in response to 5% MBBP treat group vs. model group. **Table S3.** Differential metabolites in response to 10% MBBP treat group vs. model group. **Table S4.** Differential metabolites in response to 20% MBBP treat group vs. model group. (DOCX 57 kb)

## Abbreviations

ANOVA: Analysis of variance
AST: Aspartate amino-transferase
BSTFA: Bis-(trimethylsilyl)-trifluoroacetamide
CAT: Catalase
CHE: Cholinesterase
CHOL: Total cholesterol
CPT1: Carnitine palmitoyl transferase I
Crea: Creatinine
FC: Fold change
G6Pase: Glucose-6-phosphatase
GC-MS: Gas chromatography-mass spectrometry
GLK: Glucokinase
GSH-Px: Glutathione peroxidase
HDLC: High density lipoprotein cholesterol
HMGCR: HMG-CoA reductase
HOMA-IR: Homeostasis method assessment of insulin-resistant
HPLC: High Performance Liquid Chromatography
LDLC: Low density lipoprotein cholesterol
MBBP: Mulberry branch bark powder
MDA: Malondialdehyde
OPLS-DA: Orthogonal projection to latent structures-discriminant analysis
PCA: Principal component analysis
PEPCK: Phosphoenolpyruvate carboxykinase
PFK: Phosphofructokinase
SOD: Superoxide dismutase
SPF: Specific pathogen-free
STZ: Streptozotocin
TCA cycle: The tricarboxylic acid cycle, citric acid cycle
TG: Triglyceride
TMCS: Chlorotrimethylsilane
UA: Uric acid
VIP: Variable importance in the projection
