# Supplementary material for: Metabolic effects of mulberry branch bark powder on diabetic mice based on GC-MS metabolomics approach
Source: Nutr Metab (Lond). 2019 Jan 31;16:10. doi: 10.1186/s12986-019-0335-x (PMC6357361; doi:10.1186/s12986-019-0335-x)
Supplement: Supplementary file 1 — Figure S1. The effect of high fat diet, STZ injection and MBBP treatment on the body weight of mice. Figure S2. Total ion current (TIC) chromatogram of serum of mice of Normal group (a), Model group (b), 5% MBBP treat group (c), 10% MBBP treat group (d), 20% MBBP treat group (e) obtained from GC-MS analysis. (DOCX 589 kb) [file 12986_2019_335_MOESM1_ESM.docx]

Figure S1: The effect of high fat diet, STZ injection and MBBP treatment on the body weight of mice.

(a)

(b)

(c)

(d)

(e)

Figure S2: Total ion current (TIC) chromatogram of serum of mice of Normal group (a), Model group (b), 5% MBBP treat group (c), 10% MBBP treat group (d) , 20% MBBP treat group (e) obtained from GC-MS analysis.
