# Supplementary material for: Metabolic effects of mulberry branch bark powder on diabetic mice based on GC-MS metabolomics approach
Source: Nutr Metab (Lond). 2019 Jan 31;16:10. doi: 10.1186/s12986-019-0335-x (PMC6357361; doi:10.1186/s12986-019-0335-x)
Supplement: Supplementary file 2 — Table S1. Peaks identified in TIC chromatogram of serum from five groups after peak alignment. Table S2. Differential metabolites in response to 5% MBBP treat group vs. model group. Table S3. Differential metabolites in response to 10% MBBP treat group vs. model group. Table S4. Differential metabolites in response to 20% MBBP treat group vs. model group. (DOCX 57 kb) [file 12986_2019_335_MOESM2_ESM.docx]

S1. Peaks identified in TIC chromatogram of serum from five groups after peak alignment.

| **No.** | **metabolites** | **Quant Mass** | **Retention Time** |
| --- | --- | --- | --- |
| 1 | Nicotinoylglycine | 207 | 5.150516 |
| 2 | 4-Methyl-5-thiazolethanol | 215 | 5.162528 |
| 3 | Pyruvic acid | 174 | 5.198401 |
| 4 | lactic acid | 88 | 5.249745 |
| 5 | Prostaglandin E2 | 227 | 5.301643 |
| 6 | m-cresol | 165 | 5.32972 |
| 7 | glycolic acid | 88 | 5.379038 |
| 8 | Carbazole | 167 | 5.481972 |
| 9 | sarcosine | 163 | 5.509487 |
| 10 | Maleamate | 244 | 5.555818 |
| 11 | 22-Ketocholesterol | 173 | 5.570594 |
| 12 | Maleimide | 154 | 5.614178 |
| 13 | Halostachine | 102 | 5.673576 |
| 14 | alanine | 116 | 5.664235 |
| 15 | Citraconic acid | 56 | 5.718757 |
| 16 | 1-Methylhydantoin | 258 | 5.827981 |
| 17 | fumaric acid | 245 | 5.866737 |
| 18 | 2-hydroxybutanoic acid | 131 | 5.903606 |
| 19 | L-dopa | 218 | 5.969641 |
| 20 | noradrenaline | 188 | 5.994636 |
| 21 | 3-Hydroxypropionic acid | 145 | 6.01987 |
| 22 | 4-hydroxyphenylpyruvate | 139 | 6.035751 |
| 23 | Lactamide | 232 | 6.048579 |
| 24 | Aminooxyacetic acid | 220 | 6.079499 |
| 25 | threo-beta-hyrdoxyaspartate | 98 | 6.08848 |
| 26 | N-Methyl-L-glutamic acid | 98 | 6.097088 |
| 27 | 4-HYDROXYPYRIDINE | 152 | 6.098318 |
| 28 | canavanine | 146 | 6.132223 |
| 29 | N-Acetylisatin | 89 | 6.155988 |
| 30 | proline | 142 | 6.168455 |
| 31 | kyotorphin | 241 | 6.234396 |
| 32 | N-cyclohexylformamide | 139 | 6.25586 |
| 33 | Acetophenone | 152 | 6.2848 |
| 34 | 3-hydroxybutyric acid | 88 | 6.294511 |
| 35 | sulfuric acid | 227 | 6.31392 |
| 36 | oxalic acid | 145 | 6.340635 |
| 37 | Gallic acid | 281 | 6.365395 |
| 38 | Alizarin | 216 | 6.380121 |
| 39 | 6-hydroxy caproic acid trimer | 187 | 6.42565 |
| 40 | N-Methyl-DL-alanine | 130 | 6.445696 |
| 41 | Itaconic acid | 215 | 6.457487 |
| 42 | Methyl Phosphate | 241 | 6.491653 |
| 43 | Dithioerythritol | 222 | 6.51955 |
| 44 | 6-hydroxy caproic acid | 147 | 6.543529 |
| 45 | beta-Alanine | 176 | 6.70225 |
| 46 | guaiacol | 166 | 6.645606 |
| 47 | 3-Hydroxyanthranilic acid | 136 | 6.715259 |
| 48 | alpha-ketoisocaproic acid | 200 | 6.76591 |
| 49 | Phytol | 143 | 6.79712 |
| 50 | xanthosine | 281 | 6.854297 |
| 51 | 2-Hydroxyvaleric acid | 247 | 6.935069 |
| 52 | valine | 144 | 7.01226 |
| 53 | 5-Hydroxyindole-3-acetic acid | 229 | 7.024643 |
| 54 | Pipecolinic acid | 156 | 7.062814 |
| 55 | 6-Aminopenicillanic acid | 203 | 7.072503 |
| 56 | 2-Hydroxybiphenyl | 227 | 7.114705 |
| 57 | 2-keto-isovaleric acid | 186 | 7.123874 |
| 58 | malonic acid | 217 | 7.178298 |
| 59 | D-alanyl-D-alanine | 116 | 7.237059 |
| 60 | 4-hydroxybutyrate | 233 | 7.29434 |
| 61 | Phytanic acid | 233 | 7.345616 |
| 62 | urea | 96 | 7.455277 |
| 63 | benzoic acid | 179 | 7.542684 |
| 64 | mannitol | 231 | 7.702857 |
| 65 | histidine | 154 | 7.72946 |
| 66 | 20alpha-Hydroxycholesterol | 201 | 7.744761 |
| 67 | Ethanolamine | 174 | 7.770557 |
| 68 | glycerol | 205 | 7.832729 |
| 69 | phosphate | 301 | 7.862313 |
| 70 | epsilon-Caprolactam | 244 | 7.895748 |
| 71 | pyridoxine | 281 | 7.913365 |
| 72 | Menthone | 96 | 7.928747 |
| 73 | creatine degr | 147 | 7.948708 |
| 74 | O-acetylserine | 116 | 7.984912 |
| 75 | 2-mercaptoethanesulfonic acid | 181 | 8.031756 |
| 76 | 5-Hydroxyindole-2-carboxylic acid | 231 | 8.095718 |
| 77 | Isoleucine | 158 | 8.166036 |
| 78 | L-Allothreonine | 117 | 8.177357 |
| 79 | Methoxamedrine | 116 | 8.217183 |
| 80 | N-formyl-L-methionine | 233 | 8.365644 |
| 81 | glycine | 174 | 8.39272 |
| 82 | 4-hydroxy-3-methoxycinnamaldehyde | 241 | 8.397351 |
| 83 | 5-Methoxypsoralen | 188 | 8.452223 |
| 84 | N-ethylmaleamic acid | 215 | 8.436297 |
| 85 | succinic acid | 247 | 8.497114 |
| 86 | Adipamide | 207 | 8.571106 |
| 87 | N-Acetyltryptophan | 130 | 8.5905 |
| 88 | D-Glyceric acid | 189 | 8.737757 |
| 89 | 2-Amino-2-norbornanecarboxylic acid | 108 | 8.836223 |
| 90 | uracil | 241 | 8.889691 |
| 91 | Analyte 213 | 152 | 8.983942 |
| 92 | nicotinamide | 179 | 9.004353 |
| 93 | pyrogallol | 80 | 9.022156 |
| 94 | 1-Hydroxyanthraquinone | 281 | 9.183156 |
| 95 | serine | 204 | 9.267535 |
| 96 | Homocystine | 241 | 9.280291 |
| 97 | Pelargonic acid | 215 | 9.332748 |
| 98 | norvaline | 246 | 9.376134 |
| 99 | D-erythro-sphingosine | 204 | 9.448127 |
| 100 | 3-Cyanoalanine | 141 | 9.463154 |
| 101 | Analyte 243 | 215 | 9.585764 |
| 102 | 1,2-Cyclohexanedione | 109 | 9.673008 |
| 103 | threonine | 220 | 9.725151 |
| 104 | Analyte 252 | 191 | 9.831634 |
| 105 | thymine | 255 | 9.919326 |
| 106 | 1-Indanol | 191 | 10.14387 |
| 107 | aspartic acid | 102 | 10.18014 |
| 108 | hydrocinnamic acid | 61 | 10.24619 |
| 109 | 2,4-diaminobutyric acid | 218 | 10.39295 |
| 110 | Elaidic acid | 97 | 10.43432 |
| 111 | 5-Methoxytryptamine | 174 | 10.58877 |
| 112 | 2-Deoxytetronic acid | 233 | 10.6056 |
| 113 | D-Altrose | 201 | 10.66155 |
| 114 | indole-3-acetamide | 87 | 10.80687 |
| 115 | 8-Aminocaprylic acid | 168 | 10.87493 |
| 116 | alpha-Ecdysone | 171 | 10.93075 |
| 117 | 2-amino-2-methylpropane-1,3-diol | 218 | 10.94455 |
| 118 | L-Threose | 281 | 11.10085 |
| 119 | 1,3-diaminopropane | 174 | 11.12752 |
| 120 | O-phosphonothreonine | 71 | 11.27471 |
| 121 | Dihydroxyacetone | 103 | 11.29728 |
| 122 | Aminomalonic acid | 218 | 11.32217 |
| 123 | Melatonin | 232 | 11.2213 |
| 124 | iminodiacetic acid | 232 | 11.33747 |
| 125 | Glucose-1-phosphate | 217 | 11.39467 |
| 126 | Norleucine | 158 | 11.42057 |
| 127 | N-alpha-Acetyl-L-ornithine | 168 | 11.49566 |
| 128 | N-Carbamylglutamate | 216 | 11.50169 |
| 129 | 2,6-Diaminopimelic acid | 200 | 11.58639 |
| 130 | Analyte 319 | 285 | 11.64292 |
| 131 | 5,6-dihydrouracil | 58 | 11.65577 |
| 132 | L-Malic acid | 233 | 11.6944 |
| 133 | Threitol | 217 | 11.92912 |
| 134 | 5-Aminovaleric acid | 191 | 12.01732 |
| 135 | putrescine | 174 | 12.05044 |
| 136 | methionine | 176 | 12.30727 |
| 137 | oxoproline | 258 | 12.33598 |
| 138 | Ethyl cinnamate | 247 | 12.38171 |
| 139 | Carbobenzyloxy-L-leucine degr1 | 185 | 12.40922 |
| 140 | 3-hydroxy-L-proline | 230 | 12.45855 |
| 141 | Analyte 353 | 258 | 12.48172 |
| 142 | glutamic acid | 246 | 12.50458 |
| 143 | L-glutamic acid | 314 | 12.59691 |
| 144 | DL-Anabasine | 254 | 12.69538 |
| 145 | creatine | 314 | 12.99736 |
| 146 | Indolelactate | 202 | 13.04185 |
| 147 | L-cysteine | 218 | 13.04887 |
| 148 | Threonic acid | 205 | 13.14558 |
| 149 | N-acetyl-L-aspartic acid | 202 | 13.3963 |
| 150 | alpha-ketoglutaric acid | 247 | 13.47277 |
| 151 | phosphomycin | 211 | 13.88123 |
| 152 | indole-3-acetic acid | 183 | 14.05043 |
| 153 | hexadecane | 57 | 14.0729 |
| 154 | 4-Hydroxyquinazoline | 217 | 14.21929 |
| 155 | ornithine | 142 | 14.30207 |
| 156 | phenylalanine | 192 | 14.48778 |
| 157 | 4-Hydroxybenzoic acid | 200 | 14.55771 |
| 158 | Lyxonic acid, 1,4-lactone | 174 | 14.66765 |
| 159 | O-Phosphoserine | 283 | 14.78173 |
| 160 | beta-Mannosylglycerate | 204 | 14.98957 |
| 161 | caprylic acid | 259 | 15.08907 |
| 162 | lauric acid | 257 | 15.09931 |
| 163 | ribose | 103 | 15.27802 |
| 164 | Lyxose | 103 | 15.14061 |
| 165 | taurine | 174 | 15.29894 |
| 166 | asparagine | 231 | 15.3931 |
| 167 | threo-beta-hydroxyaspartate | 221 | 15.59635 |
| 168 | 3-hydroxy-3-methylglutaric acid | 247 | 15.70278 |
| 169 | Analyte 456 | 314 | 15.78791 |
| 170 | Thymol | 207 | 15.97308 |
| 171 | Methyl Palmitoleate | 97 | 16.03705 |
| 172 | hydroxyurea | 71 | 16.12814 |
| 173 | 4-aminobutyric acid | 233 | 16.2097 |
| 174 | alpha-Aminoadipic acid | 128 | 16.37429 |
| 175 | xylitol | 217 | 16.47737 |
| 176 | 3,5-Dihydroxyphenylglycine | 182 | 16.48741 |
| 177 | D-Arabitol | 117 | 16.54103 |
| 178 | fucose | 117 | 16.79764 |
| 179 | 2,3-Dimethylsuccinic acid | 228 | 16.82157 |
| 180 | Gluconic lactone | 217 | 16.83537 |
| 181 | beta-hydroxypyruvate | 233 | 16.92531 |
| 182 | Orotic acid | 254 | 16.95669 |
| 183 | 3,6-Anhydro-D-galactose | 199 | 17.10569 |
| 184 | Analyte 513 | 82 | 17.4114 |
| 185 | glutamine | 156 | 17.5379 |
| 186 | O-Phosphorylethanolamine | 217 | 17.6923 |
| 187 | trans,trans-Muconic acid | 227 | 17.72187 |
| 188 | naphthalene | 128 | 17.75164 |
| 189 | azelaic acid | 152 | 18.03982 |
| 190 | 1-Kestose | 217 | 18.18042 |
| 191 | citric acid | 183 | 18.47586 |
| 192 | citrulline | 256 | 18.5939 |
| 193 | Analyte 559 | 160 | 18.72193 |
| 194 | Analyte 563 | 255 | 18.8259 |
| 195 | cis-gondoic acid | 283 | 18.84398 |
| 196 | Hippuric acid | 233 | 18.88126 |
| 197 | Myristic Acid | 181 | 19.09648 |
| 198 | 1,5-Anhydroglucitol | 259 | 19.10536 |
| 199 | quinic acid | 255 | 19.25413 |
| 200 | Dioctyl phthalate | 149 | 19.28578 |
| 201 | Analyte 581 | 285 | 19.37822 |
| 202 | Tagatose | 103 | 19.53304 |
| 203 | glucose | 247 | 19.68624 |
| 204 | Galactinol | 204 | 19.94899 |
| 205 | Analyte 602 | 72 | 20.02068 |
| 206 | galactose | 57 | 20.17915 |
| 207 | Erythrose | 259 | 20.27304 |
| 208 | Analyte 607 | 299 | 20.36338 |
| 209 | D-Talose | 205 | 20.41388 |
| 210 | lysine | 174 | 20.53452 |
| 211 | Melezitose | 362 | 20.58263 |
| 212 | methyl hexadecanoate | 87 | 20.63112 |
| 213 | glucuronic acid | 160 | 20.7379 |
| 214 | tyrosine | 218 | 20.81004 |
| 215 | Analyte 629 | 246 | 20.91506 |
| 216 | 4-Hydroxy-3-methoxybenzyl alcohol | 254 | 20.95845 |
| 217 | pentadecanoic acid | 232 | 21.03547 |
| 218 | cycloleucine | 156 | 21.0687 |
| 219 | Isopropyl-beta-D-thiogalactopyranoside | 217 | 21.17545 |
| 220 | trans-3,5-Dimethoxy-4-hydroxycinnamaldehyde | 246 | 21.32247 |
| 221 | L-kynurenine | 220 | 21.33625 |
| 222 | maltose | 217 | 21.61838 |
| 223 | 1-Aminocyclopropanecarboxylic acid | 187 | 21.81259 |
| 224 | ribitol | 220 | 21.8619 |
| 225 | phytosphingosine | 204 | 21.91851 |
| 226 | gluconic acid | 229 | 21.97333 |
| 227 | palmitic acid | 314 | 22.21153 |
| 228 | Galactonic acid | 205 | 22.23943 |
| 229 | Allo-inositol | 191 | 22.49252 |
| 230 | Sophorose | 204 | 22.5573 |
| 231 | androsterone | 270 | 22.63371 |
| 232 | Cysteinylglycine | 172 | 23.05196 |
| 233 | Allantoic acid | 259 | 23.23408 |
| 234 | N-Acetyl-beta-D-mannosamine | 57 | 23.30246 |
| 235 | Analyte 699 | 82 | 23.35236 |
| 236 | myo-inositol | 217 | 23.48811 |
| 237 | daidzein | 383 | 23.50843 |
| 238 | phloroglucinol | 180 | 23.56543 |
| 239 | uric acid | 199 | 23.65875 |
| 240 | heptadecanoic acid | 132 | 23.82148 |
| 241 | methyl octadecanoate | 255 | 24.07925 |
| 242 | cis-Phytol | 143 | 24.1627 |
| 243 | Octadecanol | 156 | 24.50062 |
| 244 | tetracosane | 57 | 24.6038 |
| 245 | Analyte 756 | 82 | 24.81575 |
| 246 | erythrose 4-phosphate | 283 | 24.98483 |
| 247 | Levoglucosan | 204 | 25.05638 |
| 248 | tryptophan | 202 | 25.12855 |
| 249 | trehalose-6-phosphate | 255 | 25.15026 |
| 250 | linoleic acid | 136 | 25.1619 |
| 251 | oleic acid | 222 | 25.24298 |
| 252 | sucrose | 217 | 25.30418 |
| 253 | Atrazine-2-hydroxy | 131 | 25.51143 |
| 254 | stearic acid | 117 | 25.57191 |
| 255 | spermidine | 144 | 25.6458 |
| 256 | sorbose | 225 | 25.72416 |
| 257 | dl-p-Hydroxyphenyllactic acid | 179 | 25.75516 |
| 258 | Sedoheptulose | 204 | 25.93263 |
| 259 | 5-Methoxyindole-3-acetic acid | 58 | 26.0168 |
| 260 | Analyte 809 | 82 | 26.05602 |
| 261 | glucose-6-phosphate | 218 | 26.09279 |
| 262 | 1,4-Dihydroxy-2-naphthoic acid | 246 | 26.31996 |
| 263 | Gentiobiose | 217 | 26.53483 |
| 264 | ribose-5-phosphate | 255 | 26.648 |
| 265 | linolenic acid | 106 | 26.88069 |
| 266 | lactulose | 184 | 26.9105 |
| 267 | arachidonic acid | 108 | 26.94906 |
| 268 | phloretin | 342 | 27.28636 |
| 269 | D-Fructose 1,6-bisphosphate | 301 | 27.30998 |
| 270 | methyl dodecanoate | 138 | 27.40405 |
| 271 | uridine | 132 | 27.61767 |
| 272 | Albendazole | 58 | 27.73624 |
| 273 | fructose | 103 | 27.87777 |
| 274 | saccharopine | 314 | 27.89082 |
| 275 | 2-Monoolein | 129 | 27.96907 |
| 276 | 2-Indanone | 205 | 28.0091 |
| 277 | Linoleic acid methyl ester | 136 | 28.1022 |
| 278 | maltotriose | 204 | 28.1329 |
| 279 | Methyl-beta-D-galactopyranoside | 204 | 28.38102 |
| 280 | 1-Hydroxy-2-naphthoic acid | 215 | 28.41239 |
| 281 | Glucoheptonic acid | 259 | 28.80738 |
| 282 | 1-Monopalmitin | 371 | 28.82849 |
| 283 | Behenic acid | 132 | 29.19641 |
| 284 | Phenylacetamide | 92 | 29.73675 |
| 285 | trehalose | 191 | 29.79818 |
| 286 | Monoolein | 129 | 29.86731 |
| 287 | sorbitol | 166 | 30.00337 |
| 288 | Monostearin | 57 | 30.02412 |
| 289 | 5-Aminoimidazole-4-carboxamide | 142 | 30.03311 |
| 290 | 4-Hydroxyphenylethanol | 179 | 30.09168 |
| 291 | Thioctamide | 131 | 30.27276 |
| 292 | Lignoceric acid | 140 | 30.38268 |
| 293 | Carbobenzyloxyglycine | 82 | 30.39281 |
| 294 | Analyte 997 | 112 | 30.4172 |
| 295 | 2-deoxy-D-glucose | 256 | 30.57776 |
| 296 | N-methylaniline | 106 | 30.84117 |
| 297 | Aniline-o-sulfonic acid | 82 | 30.86587 |
| 298 | Dodecanol | 112 | 31.04896 |
| 299 | biotin | 241 | 31.20375 |
| 300 | 2-Deoxy-D-galactose | 247 | 31.24501 |
| 301 | 2-Furoic Acid | 97 | 31.32884 |
| 302 | Carnitine | 106 | 31.5451 |
| 303 | beta-Glycerophosphoric acid | 243 | 31.66107 |
| 304 | D-(glycerol 1-phosphate) | 243 | 31.66872 |
| 305 | Carbobenzyloxy-L-leucine degr3 | 112 | 31.75304 |
| 306 | Cholestan-3beta-ol | 215 | 31.94684 |
| 307 | squalene | 177 | 32.17383 |
| 308 | Analyte 1056 | 106 | 32.34395 |
| 309 | Zymosterol | 255 | 32.35865 |
| 310 | 1-Hexadecanol | 82 | 32.37895 |
| 311 | alpha-Tocopherol | 112 | 32.54636 |
| 312 | L-Gulonolactone | 207 | 32.64446 |
| 313 | cholesterol | 255 | 32.70941 |
| 314 | Phosphoglycolic acid | 82 | 33.30589 |
| 315 | N-epsilon-Acetyl-L-lysine | 126 | 33.45584 |
| 316 | 1,3-Cyclohexanedione | 106 | 33.58117 |
| 317 | 4-Hydroxybenzyl cyanide | 191 | 33.86094 |
| 318 | Mandelonitrile | 255 | 33.98078 |
| 319 | palmitoleic acid | 311 | 34.34359 |
| 320 | Analyte 1111 | 82 | 34.40284 |
| 321 | β-Sitosterol | 255 | 34.62803 |

Table S2. Differential metabolites in response to 5% MBBP treat group vs. model group.

| No | Metabolite | VIP | t-test P | FC | Trend |
| --- | --- | --- | --- | --- | --- |
| 1 | Isopropyl-beta-D-thiogalactopyranoside | 1.656 | 0.00391 | 0.645 | Down |
| 2 | Maltose | 1.772 | 0.00119 | 0.621 | Down |
| 3 | Sorbitol | 1.942 | 0.00012 | 0.525 | Down |
| 4 | D-(glycerol 1-phosphate) | 1.745 | 0.00022 | 0.492 | Down |
| 5 | 3,6-Anhydro-D-galactose | 1.823 | 0.0005 | 0.659 | Down |
| 6 | 1,5-anhydroglucitol | 1.212 | 0.03201 | 0.482 | Down |
| 7 | Erythrose | 1.254 | 0.01989 | 4.378 | Up |
| 8 | Gluconic lactone | 2.082 | 8.6E-09 | 15.577 | Up |
| 9 | Galactose | 1.687 | 0.0158 | 10.922 | Up |
| 10 | 3-hydroxy-3-methylglutaric acid | 1.061 | 0.02006 | 0.431 | Down |
| 11 | Palmitoleic acid | 1.481 | 0.00039 | 0.210 | Down |
| 12 | Lauric acid | 1.985 | 8.4E-06 | 1.595 | Up |
| 13 | Linoleic acid | 2.137 | 6.7E-08 | 1.667 | Up |
| 14 | Linoleic acid methyl ester | 1.345 | 0.01638 | 2.307 | Up |
| 15 | Zymosterol | 1.005 | 1.2E-06 | 3.914 | Up |
| 16 | Hydrocinnamic acid | 1.545 | 0.01536 | 3.112 | Up |
| 17 | Arachidonic acid | 2.189 | 1.8E-11 | 4.392 | Up |
| 18 | Cholestan-3beta-ol | 1.466 | 0.00522 | 0.543 | Down |
| 19 | Beta-Alanine | 2.178 | 5.2E-08 | 0.453 | Down |
| 20 | Ornithine | 1.791 | 0.00031 | 0.651 | Down |
| 21 | 4-hydroxyphenylethanol | 2.222 | 1.8E-08 | 0.552 | Down |
| 22 | Hippuric acid | 2.073 | 1.3E-07 | 12.374 | Up |
| 23 | Oxalic acid | 2.031 | 4.3E-05 | 0.596 | Down |
| 24 | Albendazole | 1.547 | 0.00849 | 2.862 | Up |
| 25 | O-phosphorylethanolamine | 1.469 | 0.01343 | 0.527 | Down |
| 26 | Maleimide | 1.414 | 0.00033 | 0.289 | Down |
| 27 | L-cysteine | 1.984 | 4.5E-05 | 0.597 | Down |
| 28 | Pentadecanoic acid | 1.839 | 0.0029 | 6.175 | Up |
| 29 | Menthone | 1.747 | 0.00382 | 0.146 | Down |
| 30 | Iminodiacetic acid | 1.623 | 0.01342 | 9.189 | Up |
| 31 | Analyte 756 | 1.598 | 0.01954 | 0.181 | Down |
| 32 | Phytosphingosine | 1.535 | 0.01264 | 10.471 | Up |
| 33 | 2,6-Diaminopimelic acid | 1.502 | 0.00067 | 0.470 | Down |
| 34 | 4-Hydroxy-3-methoxybenzyl alcohol | 1.491 | 0.0217 | 0.000 | Down |
| 35 | Aminomalonic acid | 1.407 | 0.01758 | 1.942 | Up |
| 36 | 4-Hydroxybenzoic acid | 1.361 | 0.03766 | 13.088 | Up |
| 37 | 1,3-cyclohexanedione | 1.332 | 0.04761 | 1.967 | Up |
| 38 | 2-Hydroxyvaleric acid | 1.310 | 0.01927 | 0.396 | Down |
| 39 | N-ethylmaleamic acid | 1.287 | 0.03246 | 0.168 | Down |
| 40 | N-Methyl-L-glutamic acid | 1.162 | 0.02366 | 1.855 | Up |
| 41 | Xylitol | 1.141 | 0.04482 | 8.193 | Up |
| 42 | Phloretin | 1.138 | 0.0013 | 2.638 | Up |
| 43 | Beta-Mannosylglycerate | 1.118 | 0.04617 | 3.745 | Up |

Table S3. Differential metabolites in response to 10% MBBP treat group vs. model group

| No | Metabolite | VIP | T-test P | FC | Trend |
| --- | --- | --- | --- | --- | --- |
| 1 | Sophorose | 2.062 | 3.57031E-06 | 0.458 | Down |
| 2 | Isopropyl-beta-D-thiogalactopyranoside | 2.066 | 3.66042E-06 | 0.446 | Down |
| 3 | maltose | 2.102 | 1.22867E-06 | 0.445 | Down |
| 4 | sorbitol | 1.289 | 4.81195E-06 | 0.229 | Down |
| 5 | fructose | 1.612 | 9.16749E-05 | 0.233 | Down |
| 6 | Tagatose | 1.524 | 0.006080064 | 0.655 | Down |
| 7 | sucrose | 1.883 | 0.00036167 | 1.795 | Up |
| 8 | Erythrose | 2.276 | 6.86633E-08 | 7.817 | Up |
| 9 | Gluconic lactone | 2.111 | 1.59551E-09 | 24.361 | Up |
| 10 | galactose | 2.088 | 8.14303E-06 | 20.043 | Up |
| 11 | beta-Glycerophosphoric acid | 1.416 | 0.024361515 | 0.261 | Down |
| 12 | ribitol | 2.025 | 5.1367E-06 | 0.463 | Down |
| 13 | ribose | 2.338 | 3.6262E-13 | 0.000 | Down |
| 14 | sorbose | 1.807 | 0.000342227 | 0.539 | Down |
| 15 | 2-keto-isovaleric acid | 1.986 | 4.39714E-05 | 1.641 | Up |
| 16 | linolenic acid | 1.961 | 3.07266E-05 | 0.641 | Down |
| 17 | linoleic acid | 1.898 | 0.000221377 | 1.577 | Up |
| 18 | Linoleic acid methyl ester | 1.297 | 0.013025128 | 2.378 | Up |
| 19 | arachidonic acid | 2.177 | 1.79261E-08 | 4.726 | Up |
| 20 | Cholestan-3beta-ol | 1.869 | 0.000187533 | 0.415 | Down |
| 21 | heptadecanoic acid | 1.720 | 0.001076684 | 0.642 | Down |
| 22 | cycloleucine | 1.387 | 0.000157427 | 0.271 | Down |
| 23 | beta-Alanine | 1.870 | 4.44939E-05 | 0.581 | Down |
| 24 | citrulline | 1.870 | 8.22713E-05 | 1.877 | Up |
| 25 | 4-Hydroxyphenylethanol | 2.159 | 6.42762E-09 | 0.409 | Down |
| 26 | Hippuric acid | 1.440 | 0.016120654 | 7.682 | Up |
| 27 | oxalic acid | 1.921 | 0.000179713 | 0.650 | Down |
| 28 | Pipecolinic acid | 2.048 | 1.84212E-05 | 1.929 | Up |
| 29 | taurine | 1.907 | 0.000475994 | 1.881 | Up |
| 30 | O-Phosphorylethanolamine | 1.863 | 0.001658868 | 0.351 | Down |
| 31 | Maleimide | 1.107 | 0.001033667 | 0.426 | Down |
| 32 | Allantoic acid | 1.469 | 0.008623292 | 1.732 | Up |
| 33 | Menthone | 1.177 | 0.009113247 | 0.269 | Down |
| 34 | 2-amino-2-methylpropane-1,3-diol | 1.313 | 0.015872057 | 0.467 | Down |
| 35 | norvaline | 1.590 | 0.00032086 | 1.930 | Up |
| 36 | Glucoheptonic acid | 1.727 | 0.016346998 | 0.204 | Down |
| 37 | threo-beta-hyrdoxyaspartate | 1.697 | 0.026209272 | 4.537 | Up |
| 38 | alpha-ketoisocaproic acid | 1.525 | 0.017568106 | 1.951 | Up |
| 39 | 2-Monoolein | 1.455 | 0.016010699 | 2.083 | Up |
| 40 | 4-hydroxyphenylpyruvate | 1.408 | 0.025417938 | 7.804 | Up |
| 41 | Melatonin | 1.382 | 0.022790286 | 1.699 | Up |
| 42 | hydroxyurea | 1.346 | 0.005976882 | 2.592 | Up |
| 43 | beta-hydroxypyruvate | 1.241 | 0.022919388 | 0.605 | Down |

Table S4. Differential metabolites in response to 20% MBBP treat group vs. model group.

| No | Metabolite | VIP | t-test P | FC | Trend |
| --- | --- | --- | --- | --- | --- |
| 1 | Sophorose | 1.847 | 2.99E-05 | 0.474 | Down |
| 2 | Isopropyl-beta-D-thiogalactopyranoside | 1.849 | 4.67E-05 | 0.474 | Down |
| 3 | maltose | 1.856 | 2.56E-05 | 0.453 | Down |
| 4 | Tagatose | 1.621 | 0.002799 | 0.631 | Down |
| 5 | fucose | 1.617 | 0.001928 | 1.566 | Up |
| 6 | maltotriose | 1.246 | 0.022431 | 2.373 | Up |
| 7 | sucrose | 1.807 | 0.007039 | 2.271 | Up |
| 8 | Erythrose | 2.252 | 7.75E-09 | 9.582 | Up |
| 9 | Gluconic lactone | 2.078 | 8.04E-07 | 42.581 | Up |
| 10 | galactose | 1.440 | 0.003685 | 15.463 | Up |
| 11 | beta-Glycerophosphoric acid | 1.212 | 0.034755 | 0.329 | Down |
| 12 | gluconic acid | 1.389 | 0.038308 | 0.470 | Down |
| 13 | ribitol | 1.812 | 4.75E-05 | 0.486 | Down |
| 14 | ribose | 1.990 | 0.001082 | 0.193 | Down |
| 15 | citric acid | 1.851 | 0.000291 | 1.596 | Up |
| 16 | L-Malic acid | 1.704 | 0.000468 | 1.513 | Up |
| 17 | 2-keto-isovaleric acid | 1.995 | 3.4E-05 | 1.779 | Up |
| 18 | linolenic acid | 1.824 | 7.27E-05 | 0.647 | Down |
| 19 | linoleic acid | 2.148 | 1.93E-07 | 1.949 | Up |
| 20 | Linoleic acid methyl ester | 1.270 | 0.026485 | 2.181 | Up |
| 21 | arachidonic acid | 2.112 | 4.38E-07 | 4.618 | Up |
| 22 | Cholestan-3beta-ol | 1.359 | 0.00422 | 0.569 | Down |
| 23 | heptadecanoic acid | 1.321 | 0.024421 | 0.486 | Down |
| 24 | cycloleucine | 1.626 | 0.004641 | 0.648 | Down |
| 25 | citrulline | 2.019 | 3.1E-06 | 2.231 | Up |
| 26 | saccharopine | 1.506 | 0.009666 | 1.835 | Up |
| 27 | 4-Hydroxyphenylethanol | 2.047 | 9.4E-09 | 0.379 | Down |
| 28 | Hippuric acid | 1.732 | 0.000465 | 13.348 | Up |
| 29 | Pipecolinic acid | 2.003 | 3.2E-05 | 2.160 | Up |
| 30 | taurine | 2.183 | 2.37E-07 | 2.495 | Up |
| 31 | O-Phosphorylethanolamine | 1.829 | 0.001413 | 0.336 | Down |
| 32 | Maleimide | 1.560 | 0.000716 | 0.310 | Down |
| 33 | 1-Aminocyclopropanecarboxylic acid | 1.652 | 0.004076 | 1.555 | Up |
| 34 | Allantoic acid | 1.757 | 0.000404 | 2.395 | Up |
| 35 | 4-Hydroxybenzoic acid | 1.229 | 0.001847 | 13.805 | Up |
| 36 | phloretin | 1.130 | 0.001811 | 3.525 | Up |
| 37 | 4-hydroxybutyrate | 1.679 | 0.002397 | 0.546 | Down |
| 38 | m-cresol | 1.297 | 0.004123 | 1.612 | Up |
| 39 | Carbazole | 1.750 | 0.004173 | 0.000 | Down |
| 40 | L-dopa | 1.099 | 0.045886 | 1.725 | Up |
